# Supplementary material for: A participatory approach to move towards a One Health surveillance system for anthrax in Burkina Faso
Source: PLoS One. 2024 Jun 5;19(6):e0304872. doi: 10.1371/journal.pone.0304872 (PMC11152267; doi:10.1371/journal.pone.0304872)
Supplement: Attachment — Submitted filename: Response to reviewers.pdf [file pone.0304872.s004.pdf]

Responses to reviewer comments

REVIEWER 1

Introduction

The authors don't mention the presence of a One Health National Platform in Burkina Faso. So, among the participant invited, this stakeholder is don't take account. Even if another paper is already published on the current involved stakeholders in the anthrax surveillance, it is necessary to remind briefly here.
We have mentioned the platform in the introduction of the revised manuscript (see lines 51 and 52)

Material and methods

In the participatory process, it is necessary to implement the system and use results to correct the system. That can constitute the fifth step.
In participatory planning, the participatory process aims at the co-construction of an action plan for the establishment of the desired One Health surveillance system. The implementation of the action plan is not part of the process.
However, it is valuable to evaluate the implementation of the action plan and its effects, as part of the evaluation process of the participatory process. We developed this point in lines 380-386.

When you say Central and local authorities, it is necessary to precise the level of the positioning (National, provincial, departmental, village, etc.). In the table 2, at the points 5.2 & 5.3, you mentioned Regional, provincial and local level. What "local level" means?
By local authorities, we refer to State agents working in the field, i.e the ones who are at the frontline for the observation of the health events. Depending on their supervising ministry, their area of action differs but their level is below the provincial one. So, we cannot specify a generic administrative level suitable for all the local authorities. We have revised the manuscript to clarify this point, as followed: "Local authorities refer to field actors working for ministries in charge of public health, animal health and wildlife conservation" (see lines 96-97).

For each institution, what kind of personal (head or agent) was took account? That is important in the future implementation of the changes' actions.
The target audience was technical agents directly involved in surveillance activities because we assumed that they would be the most capable to share knowledge about surveillance and to identify necessary changes to improve integrated surveillance. The drawback of this approach is that the changes and actions defined are not communicated and bought in by the people who have the power to implement them. This is a limitation of our study that we discussed lines 296-299.

From the Table 1, can you explain the reason of the total or partial absence of some stakeholder? What were the financial supported for the participants? That can explain also the quality of the participants.
The two missing participants were engaged in other official duties on these days. One missed only one on the three days (the 2nd day), and this suggests his interest for the workshop.
Participants were compensated for their travel expenses and were offered lunch for the three days. Those travelling from provincial areas were compensated for accommodation and food expenses.
Providing financial support may also have a negative impact on the quality of the participants, as some people may be attracted by the money, more than by the interest for the topic. In our study, we did not use financial support to motivate people but we created a climate of trust with participants with whom we had interacted prior the workshop. We also paid attention to the date and location of the workshop to improve the likelihood of their availability and attendance (see lines 97-99).

Lines 98 – 99: “appropriate participative tools” used can be cited here. Tools used are so important as the quality of facilitators.
The participative tools used and the way they were used are described at each step of the participatory process in the Material and Method part.

Lines 104 – 106: The evaluation is an essential part of the process building. The evaluation method must be presented in this paper.
Because of word limitation, we cannot present the evaluation plan in this paper but we intend to publish it in a separate paper. However, we have added a short description of it: “Qualitative and semi-quantitative indicators were measured based on data collected before, during and after the workshop, using different methods (questionnaire, semi-structured interviews, workshop observation)” (see lines 111-113).

Figure 2: Why different boxes has the same number? The numbers must be different and classified. Review also the abscissa axis texts: What mean T0? T0 + 10 ***ans****?*
The figure has been updated following the recommendations and a legend added. Additionally, “ans” has been changed for “years”.

RESULTS

Line 181: notification of suspected or confirmed cases? Need the precision. In the lines 201 to 207, it is mentioned the suspected cases.
The notification of suspected cases must be followed by an investigation to confirm or infirm them. We have updated the wording of the title to be consistent with the changes identified (see line 213).

How, the time 10 years had been defined? Is it the time of evaluation of the impact?
The time step was defined on the basis of our knowledge of the surveillance context in Burkina Faso. It seemed to us to be the most realistic for setting up an integrated surveillance system. Far enough away for participants to be able to propose far-reaching changes. Not too far in the future for them to be able to project themselves. This timeframe was not questioned by the participants, suggesting that they were also comfortable with this choice. We have made this precision in the revised manuscript (see lines 123-125).

Figure 3, 4, 5, 6 & 7. Review pictures’ resolutions for a best reading
Figures resolution has been improved and checked using the PLOS tool. Please note that when the PLOS system builds the PDF which is sent to reviewers, the resolution is decreased. To see the original file, you need to click on “Click here to access/download xxx” at the top of the page where the figure is displayed.

Table 2: Repeat the columns’ titles in each page can help for the best reading du to the width of this table.
The table has been updated to meet this request.

In the point 7 of the table 2, keep the original denomination of all services: ZATE, CSPS, PV, PF, etc.)
The table has been updated in the revised version of the manuscript to take into consideration this comment.

The action plan presented in the table 2 is not planed in time. How that can be reached before the 10 years?

During the workshop, we only worked in defining the staring time and time period for the first action of each change. We did not include it in the manuscript for space reason and because we consider that this information was not crucial in a scientific article.

At this stage of your study, is it possible to know if process succeed or not? That is not clear in the manuscript.
That depends what we mean by success. This question will be partially answered with the results of the evaluation process that will be published in a separate manuscript. Indeed, so far, only the immediate and intermediate effects of the process have been evaluated. Long-term effects, including the implementation of action plan and its impacts, have not been evaluated yet. It is important to mention that the success of a participatory process does not only rely in the results produced but also in the knowledge and mutual understanding gained by participants during the process.
We know that the action plan has not been approved yet but that it will be used to develop the upcoming surveillance action plan piloted by the national One Health Platform.
We have updated the manuscript to include some observations that suggested that the workshop was a success regarding some criteria: "The co-construction process proposed in Burkina Faso to collectively define the modalities for implementing the One Health surveillance system of anthrax appears satisfactory in terms of the results produced and the active participation of those invited. Participants expressed their satisfaction regarding the workshop organization and the results produced, and stated that they would take actions to contribute to the application of the decisions taken. They also considered that they improved their knowledge about the epidemiology of anthrax, the organization and functioning of anthrax surveillance" (see lines 369-373).

Discussion

Is it possible to evaluate the cost of the changes proposed to improve the current surveillance system? Also discuss the feasibility of this changes regarding the cost, and the financial sources.
An economic evaluation would be a strong added value to support the implementation of the action plan but it was not conducted in this study. This perspective has been added in the discussion part of the manuscript (see lines 364-365).
Allocation of appropriate financial resources has been identified a main barrier to the implementation of the action plan and this has been added in the revised manuscript (see lines 254-255).

REVIEWER 2

Title:

This can be read as a parallel system along with the existing sectoral ones. Which is not the case, refer to. figure 4. The word "current" is used in the article to talk about the sector surveillance system, however. Make it i explicit and clear that the One Health anthrax surveillance "system" is developed by leveraging the existing sector surveillance system and creating bridges between them.
We proposed to change the initial title to:
"A participatory approach to move towards a One Health surveillance system for anthrax in Burkina Faso". (See lines 1-3).
Moving towards a One Health surveillance system is not only creating bridged between sectoral components but also reinforcing sectoral surveillance performance (as underlined by participants of this study).
In the introduction, we mentioned explicitly that we were not proposing to develop a new system but to improve the current one, in line with the OH concept: "identify the changes, and associated

actions, necessary to evolve from the current situation to the desired situation" (see lines 74-76). We make it more explicit in the Materials and Methods section (see lines 83-84).

Abstract:

IS this a definition of an integrated surveillance system through One Health lens?
We use the term integration in its primary signification, which is "to bring together or incorporate (parts) into a whole". In the context of surveillance, integration refers to the fact different that surveillance components are linked together. In a One Health context, integrated surveillance brings together surveillance components that are operating in the three main sectors (when relevant): human, animal, environment.
We made the definition more precise: "Regarding epidemiological surveillance, this implies the development of integrated systems that link surveillance components operating in different domains (human, domestic animals, environment) and involving several actor networks." (see lines 20-22).

Be specific, add the type of interaction, Is it for instance information sharing?
Interaction may happen both at governance (steering, coordination, technical and scientific support) and operation levels (data collection, data management and storage, data analysis and interpretation, result communication and dissemination).
We have updated the manuscript as follow: "However, surveillance continue to operate in a very compartmentalized way, with little interaction between sectoral institutions and with the community for the governance and operation of surveillance activities." (see lines 22-25).

I suggest to rewrite this sentence. This can be read as an affirmation, which stakeholders are you referring to?
This is well documented that development and research programmes are usually designed and implemented with an insufficient consideration of the points of view of all categories of stakeholders, especially actors working at the local level.
However, in order to balance our words, we proposed to rewrite the sentence, as followed: "This is partly explained by the insufficient consideration of the local context and the late involvement of national stakeholders when developing programmes that aimed at strengthening the integration of surveillance." (See lines 25-27).

This can be read as national counterpart does not have a say. If correct, I suggest to rewrite this sentence to have a better balance
The manuscript is mentioning the influence of the external partners (through the organization and financing of workshops, seminars and trainings in particular). This does not suggest that national actors cannot raise their voice. We think that the current wordings is correct to describe this situation.

[Mutual understanding] of what? the existing systems?, if yes, be specific
We have defined this term in the introduction (lines 47-48): *understanding one another's thoughts, feelings, and perspectives)*

What about coordination and cooperation? [in addition to collaboration]
Collaboration is at the heart of the One Health concept as mentioned in most of the definitions of the concept in the literature. We use collaboration in a comprehensive way that covers also collaboration for coordinating activities and for communication. Cooperation is a very close concept to collaboration. Adding coordination and cooperation will make the text more difficult to read, without bringing crucial additional information.

Introduction

[Regarding non tangible benefits] Can be read as non-monetary. if correct, use non-monetary
Non tangible benefits, as well as tangible benefits, could be monetary and non-monetary. So non tangible is not synonymous with non-monetary.

Materials and methods

[Regarding the participants] Which quality? is related to the Knoweledge on the matter/subject?
The quality of participants is not referring to the knowledge only. It is related to the adequation between the participant characteristics (in terms of capacities, capabilities, knowledge, practices, etc.) and the category of the stakeholder he/her is representing

[Regarding participant selection], I understand, selection criteria was applied. Correct?
Yes, as described right after.

I understand, a consensus on the defined vision, correct?
In this type of approach, we are not necessarily looking for a consensus; it may sometimes be a question of compromise, the objective being that all the participants find themselves in the representation produced

evaluation matrix or evaluation plan?
This an evaluation plan

Is it for each sector? If yes, be specific.
No, it is not. It is the whole surveillance system, ie all actors involved in surveillance, whatever their sector, and interactions between them (within a sectoral surveillance component and between sectoral components). We believe that the sentences just after clarify already this point.

The next two comments were uncompleted sentences and could not be addressed:
"This can be read as an assessment (formal or not) of existing surveillance systems was con"
"Is the information flow w"

Is any identified official document used to "which actor needs to do what differently?
We did not explicitly refer to any official document to support this step. We let participants expressed changes based on their knowledge and expectations, which may have been influenced by official documents, such as international standards or national legal instruments. But, we did not ask and record for this information.
Expressing changes in terms of "which actor needs to do what differently?" is the most commonly adopted way in change-oriented approaches.

[Regarding the use of integrated surveillance], use One Health surveillance instead
We changed integrated surveillance system to One Health surveillance system, where appropriate.

Results

[Regarding the formalization of exchanges] *This can be read as there is no administrative decision of their involvement as actor. Is it correct?*

This means that there is no official document describing the terms of these exchanges. We have revised the manuscript to make this clearer: "All these changes were made with a high degree of consensus, except for the interactions linked to information sharing between field actors, which gave rise to differences of opinion. In fact, these exchanges are not formalized in an official document, …". (See line 202-206)

[Regarding changes proposed by the participants] *If there is no administrative decision/adopted formal framework or any other formal documents to streamline multisectoral collaboration, coordination and cooperation acheiving these changes might be difficult*
Is this disccused during the workshop?
The participants discussed this point. That's why they identified some changes in terms of intersectoral governance (see lines 235-238) and the need to validate and implement the action plan through the inter-ministerial platform (see lines 253-254).

Suggestion to change "A specific intersectoral plan" for "A anthrax intersectoral actionable plan (WHAT, WHEN, HOW, WHO,....) should be developed and implemented."
This paper reflects the views of the workshop participants who expressed themselves with this terminology. Consequently, we propose to keep the text as it is.

What is the added value of integrating the anthrax specific intersectoral plan into the global intersectoral plan for zoonoses
The participants thought that the plan will gain in attention if it was not staying standalone but integrated in an overarching document, making it easier to seek funding for initiatives. Moreover, some actions might be applicable for other diseases and thematic. We have updated the manuscript to mention this point (see lines 238-240).

You should propose to conduct a multisectoral After Action Review when a Anthrax event is closed
This paper reports changes and actions that were proposed by workshop participants. It is not intended to reflect the view of the researchers who facilitated the workshop.

Above, the word "report" was used. Use the same word here and accross the manuscript
We changed report (fewer occurrences) for notification in the whole manuscript.

Is the OHP invited aven as observer?
The OHP was one of the participants of the workshop, as mentioned in table 1 (national authorities at the multisectoral level).

[Regarding the lack of political will], Be more explicite? What do you mean by this?
We refer to the lack of political will to allocate resources for surveillance. We brought this precision in the manuscript: "The existing national One Health platform represents an appropriate governance framework for implementing the actions identified. However, the lack of political will to allocate more resources for surveillance" (see line 255).

Regarding request to change order in table 2.
We kept changes in the chronological order as defined by participants during the workshop. They considered that equipping agents with tablets was coming first, then updating the current forms, and finally training the agents. This can be discussed and revised at a next step of the process but for now, for ethical reason, we prefer to strictly comply with what was agreed on during the workshop.

Discussion

[Regarding the collective representation of the current situation] *be specific. of what? Sector surveillance system? This wording can be understood as an assessment was conducted. The fig 4 provides information flow within each system and the arrows the necessary bridges among the systems.*
This is a representation of the current situation of the surveillance in Burkina, in all sectors combined. We made this clearer in the revised manuscript: “It led to a collective representation of the current surveillance system and the desired surveillance system” (see lines 264-265).
We fail to see how this wording can suggest that an assessment was done. A representation is a way to model a situation, in our case, through the design of an actor diagram. Figure 4, as mentioned in the title, is a representation of the current surveillance situation (in all sectors) using an actor diagram, where actors are represented together with the interactions that exist between them (are they belonging to the same sectoral component or not).

Instead of capacity use the knowledge and experience on the matter/subject/
Capacities is not only related to knowledge and experience, but also related to representativeness and influence, as developed in the next paragraph.
We updated the manuscript as followed: “(i) the representativeness, knowledge and influence of the participants” (see lines 280-281).

Figure 1

Add s at system
There is only one system, consisting of several surveillance components; so no s is needed at system

add before changes, the words " institutional and technical " changes. Example of Institutional change: adoption of formal framework for information sharing among sector. Example of technical change: Use of koint SOPs,...........
We do not know in advance what kind of changes will be identified by participants and identified changes may not fall into the institutional and technical categories (behavioural changes for instance). As this method is intended to be generic, we prefer to keep changes without any specific qualification.

Photo 2

Add legend. Blue:.....Green:.....Orange: Arrow: …
Is the different colors represent each and the arrows the information flow within and between sectors?
This photo here is to illustrate the participatory tool that was used to support participants in the co-construction process of the current surveillance situation (material and method section). We do not expect the reader to be able to read the actor diagram here. Moreover, it is presented in the result section (with the appropriate legend).

Figure 3.

Community health workers can be read as human health worker. If it is not the case be specific.
It refers to community agents working in the human (CHW), animal (CAHWs) and environmental (ecoguard) sectors. We have changed for community agents to be more generic.

We have updated the figure with the 2 requests of rewording (effective instead of good and addition of administrative before mechanisms)
