# Supplementary material for: A participatory approach to move towards a One Health surveillance system for anthrax in Burkina Faso
Source: PLoS One. 2024 Jun 5;19(6):e0304872. doi: 10.1371/journal.pone.0304872 (PMC11152267; doi:10.1371/journal.pone.0304872)
Supplement: Attachment — Submitted filename: PONE-D-23-41971_R1.pdf [file pone.0304872.s005.pdf]

PLOS ONE

A participatory approach to move towards a One Health surveillance system for anthrax in Burkina Faso

--Manuscript Draft--

**Manuscript Number:**	PONE-D-23-41971R1
**Article Type:**	Research Article
**Full Title:**	A participatory approach to move towards a One Health surveillance system for anthrax in Burkina Faso
**Short Title:**	A participatory approach to develop a One Health surveillance system
**Corresponding Author:**	Marion Bordier<br>Centre for International Cooperation in Agricultural Development Research: CIRAD<br>Dakar, SENEGAL
**Keywords:**	anthrax; Burkina Faso; integrated; One Health; Participation; Surveillance; Zoonotic diseases
**Abstract:**	The One Health approach calls for collaboration across various sectors and different scales to improve understanding of complex health issues. Regarding epidemiological surveillance, this implies the development of integrated systems that link several surveillance components operating in different domains (human, domestic animals, environment) and involving several actor networks. However, surveillance continues to operate in a very compartmentalized way, with little interaction between sectoral institutions and with the community for the governance and operation of surveillance activities. This is partly explained by the insufficient consideration of the local context and the late involvement of national stakeholders when developing programmes that aimed at strengthening the integration of surveillance. In low- and middle-income countries in particular, there is a strong influence of external partners on the development of intersectoral programmes, including surveillance systems. In this context, we developed and implemented a participatory planning process to support stakeholders of the surveillance system of anthrax in Burkina Faso, in the definition of the One Health surveillance system they wish for and of the pathway to reach it. The workshop produced an action plan that reflects the views and perspectives of representatives of the different categories of stakeholders and beneficiaries of surveillance. In addition, the participation of stakeholders in this participatory co-construction process has also improved their knowledge and mutual understanding, fostering a climate of trust conducive to further collaboration for surveillance activities. However, the quality of the participation raises some questions over the results, and contextual factors may have influenced the process. This underlines the need to include a monitoring and evaluation plan in the process to assess its implementation and ability to produce One Health surveillance modalities that are appropriate, accepted and applied over the long term.
**Order of Authors:**	Sougrenoma Désiré Nana
	Raphaël Duboz
	Potiandi Serge Diagbouga
	Pascal Hendrikx
	Marion Bordier
**Response to Reviewers:**	All responses to editor and reviewers have been submitted through the "cover letter file" and the "response to reviewers" file.
**Additional Information:**	
**Question**	**Response**
**Financial Disclosure**<br><br>Enter a financial disclosure statement that	Yes

Powered by Editorial Manager® and ProduXion Manager® from Aries Systems Corporation

describes the sources of funding for the work included in this submission. Review the submission guidelines for detailed requirements. View published research articles from *PLOS ONE* for specific examples.<br><br>This statement is required for submission and **will appear in the published article** if the submission is accepted. Please make sure it is accurate.<br><br>**Funded studies**<br>Enter a statement with the following details:<br>• Initials of the authors who received each award<br>• Grant numbers awarded to each author<br>• The full name of each funder<br>• URL of each funder website<br>• Did the sponsors or funders play any role in the study design, data collection and analysis, decision to publish, or preparation of the manuscript?<br><br>Did you receive funding for this work?	
Please add funding details.<br>as follow-up to "**Financial Disclosure**<br><br>Enter a financial disclosure statement that describes the sources of funding for the work included in this submission. Review the submission guidelines for detailed requirements. View published research articles from *PLOS ONE* for specific examples.<br><br>This statement is required for submission and **will appear in the published article** if the submission is accepted. Please make sure it is accurate.	This work was funded in part by CIRAD, VetagroSup (the French institute for higher education and research in food, animal health, agricultural and environmental sciences) and the French Embassy in Burkina Faso.

Powered by Editorial Manager® and ProduXion Manager® from Aries Systems Corporation

**Funded studies**<br>Enter a statement with the following details:<br>• Initials of the authors who received each award<br>• Grant numbers awarded to each author<br>• The full name of each funder<br>• URL of each funder website<br>• Did the sponsors or funders play any role in the study design, data collection and analysis, decision to publish, or preparation of the manuscript?<br><br>Did you receive funding for this work?"	
Please select the country of your main research funder (please select carefully as in some cases this is used in fee calculation).<br>as follow-up to "**Financial Disclosure**<br><br>Enter a financial disclosure statement that describes the sources of funding for the work included in this submission. Review the submission guidelines for detailed requirements. View published research articles from *PLOS ONE* for specific examples.<br><br>This statement is required for submission and **will appear in the published article** if the submission is accepted. Please make sure it is accurate.<br><br>**Funded studies**<br>Enter a statement with the following details:<br>• Initials of the authors who received each award<br>• Grant numbers awarded to each author<br>• The full name of each funder<br>• URL of each funder website<br>• Did the sponsors or funders play any role in the study design, data collection and analysis, decision to publish, or preparation of the manuscript?<br><br>Did you receive funding for this work?"	BURKINA FASO - BF
**Competing Interests**	The authors have declared that no competing interests exist.

Use the instructions below to enter a competing interest statement for this submission. On behalf of all authors, disclose any competing interests that could be perceived to bias this work—acknowledging all financial support and any other relevant financial or non-financial competing interests.<br><br>This statement is required for submission and **will appear in the published article** if the submission is accepted. Please make sure it is accurate and that any funding sources listed in your Funding Information later in the submission form are also declared in your Financial Disclosure statement.<br><br>View published research articles from *PLOS ONE* for specific examples.<br><br>**NO authors have competing interests**<br><br>Enter: *The authors have declared that no competing interests exist.*<br><br>**Authors with competing interests**<br><br>Enter competing interest details beginning with this statement:<br><br>*I have read the journal's policy and the authors of this manuscript have the following competing interests: [insert competing interests here]*<br><br>* typeset	
**Ethics Statement**<br><br>Enter an ethics statement for this submission. This statement is required if the study involved:<br><br>• Human participants<br>• Human specimens or tissue<br>• Vertebrate animals or cephalopods<br>• Vertebrate embryos or tissues	This study was evaluated and validated by the ethics committee of the Ministry of Higher Education, Scientific Research and Innovation of Burkina Faso in March 2021 by deliberation n° 2021–07-161. A written informed consent to workshop participation was obtained from all participants.

• Field research<br><br>Write "N/A" if the submission does not require an ethics statement.<br><br>General guidance is provided below. Consult the submission guidelines for detailed instructions. **Make sure that all information entered here is included in the Methods section of the manuscript.**	

Powered by Editorial Manager® and ProduXion Manager® from Aries Systems Corporation

**Format for specific study types**<br><br>**Human Subject Research (involving human participants and/or tissue)**<br>• Give the name of the institutional review board or ethics committee that approved the study<br>• Include the approval number and/or a statement indicating approval of this research<br>• Indicate the form of consent obtained (written/oral) or the reason that consent was not obtained (e.g. the data were analyzed anonymously)<br><br>**Animal Research (involving vertebrate animals, embryos or tissues)**<br>• Provide the name of the Institutional Animal Care and Use Committee (IACUC) or other relevant ethics board that reviewed the study protocol, and indicate whether they approved this research or granted a formal waiver of ethical approval<br>• Include an approval number if one was obtained<br>• If the study involved *non-human primates*, add *additional details* about animal welfare and steps taken to ameliorate suffering<br>• If anesthesia, euthanasia, or any kind of animal sacrifice is part of the study, include briefly which substances and/or methods were applied<br><br>**Field Research**<br><br>Include the following details if this study involves the collection of plant, animal, or other materials from a natural setting:<br>• Field permit number<br>• Name of the institution or relevant body that granted permission	
**Data Availability**<br><br>Authors are required to make all data underlying the findings described fully available, without restriction, and from the time of publication. PLOS allows rare exceptions to address legal and ethical concerns. See the PLOS Data Policy and FAQ for detailed information.	No - some restrictions will apply

Powered by Editorial Manager® and ProduXion Manager® from Aries Systems Corporation

A Data Availability Statement describing where the data can be found is required at submission. Your answers to this question constitute the Data Availability Statement and **will be published in the article**, if accepted.<br><br>**Important:** Stating 'data available on request from the author' is not sufficient. If your data are only available upon request, select 'No' for the first question and explain your exceptional situation in the text box.<br><br>Do the authors confirm that all data underlying the findings described in their manuscript are fully available without restriction?	
**Describe where the data may be found in full sentences. If you are copying our sample text, replace any instances of XXX with the appropriate details.**<br><br>• If the data are **held or will be held in a public repository**, include URLs, accession numbers or DOIs. If this information will only be available after acceptance, indicate this by ticking the box below. For example: *All XXX files are available from the XXX database (accession number(s) XXX, XXX.*).<br>• If the data are all contained **within the manuscript and/or Supporting Information files**, enter the following: *All relevant data are within the manuscript and its Supporting Information files.*<br>• If neither of these applies but you are able to provide **details of access elsewhere**, with or without limitations, please do so. For example:<br><br>*Data cannot be shared publicly because of [XXX]. Data are available from the XXX Institutional Data Access / Ethics Committee (contact via XXX) for researchers who meet the criteria for access to confidential data.*<br><br>*The data underlying the results presented in the study are available from (include the name of the third party*	All relevant data to support the replication of this study are within the manuscript, except the name and contact details of workshop participants which remain confidential for ethical reasons.

and contact information or URL).<br>• This text is appropriate if the data are owned by a third party and authors do not have permission to share the data.<br><br>* typeset	
Additional data availability information:	

Powered by Editorial Manager® and ProduXion Manager® from Aries Systems Corporation

Revised manuscript clean

Click here to access/download;Manuscript;20240312_Co-construction_SSI_anthrax_Burkina.docx

A participatory approach to move towards a One Health surveillance system for anthrax in Burkina Faso

Sougrenoma Désiré Nana [1,2], Raphaël Duboz[1,3,4], Potiandi Serge Diagbouga[5,6], Pascal Hendrikx[7] and Marion Bordier[1,3,8*]

[1] UMR ASTRE, University of Montpellier, CIRAD, INRAE, Montpellier, France

[2] UMR ASTRE, CIRAD, Montpellier, France

[3] UMR ASTRE, CIRAD, Dakar, Senegal

[4] UMMISCO, IRD, Sorbonne University, Bondy, France

[5] Research Institute of Health Sciences, Ouagadougou, Burkina Faso

[6] Health Training and Research &Development, Ouagadougou, Burkina Faso.

[7] High Council for Food, Agriculture and Rural Areas, Paris, France.

[8] National Laboratory for Livestock and Veterinary Research, Senegalese Institute of Research in Agriculture, Dakar, Senegal

*Corresponding author

E-mail: marion.bordier@cirad.fr (MB)

Abstract

The One Health approach calls for collaboration across various sectors and different scales to improve understanding of complex health issues. Regarding epidemiological surveillance, this implies the development of integrated systems that link several surveillance components operating in different domains (human, domestic animals, environment) and involving several actor networks. However, surveillance continues to operate in a very compartmentalized way, with little interaction between sectoral institutions and with the community for the governance and operation of surveillance activities. This is partly explained by the insufficient consideration of the local context and the late

involvement of national stakeholders when developing programmes that aimed at strengthening the integration of surveillance. In low- and middle-income countries in particular, there is a strong influence of external partners on the development of intersectoral programmes, including surveillance systems. In this context, we developed and implemented a participatory planning process to support stakeholders of the surveillance system of anthrax in Burkina Faso, in the definition of the One Health surveillance system they wish for and of the pathway to reach it. The workshop produced an action plan that reflects the views and perspectives of representatives of the different categories of stakeholders and beneficiaries of surveillance. In addition, the participation of stakeholders in this participatory co-construction process has also improved their knowledge and mutual understanding, fostering a climate of trust conducive to further collaboration for surveillance activities. However, the quality of the participation raises some questions over the results, and contextual factors may have influenced the process. This underlines the need to include a monitoring and evaluation plan in the process to assess its implementation and ability to produce One Health surveillance modalities that are appropriate, accepted and applied over the long term.

Introduction

The One Health approach promotes collaboration between sectors, professions, and disciplines to improve the health of humans, animals, and ecosystems on an equitable basis [1]. The application of this concept to epidemiological surveillance is expected to result in greater epidemiological and economic performance of surveillance systems. Indeed, it is expected to improve knowledge of health events and their management while reducing the operational costs of surveillance [2-6]. Less tangible benefits may also occur, such as the establishment of mutual understanding (i.e., understanding one another's thoughts, feelings, and perspectives) and trust between actors, conducive to further and deeper collaboration [4, 7]. However, the operationalization of the principles of the One Health concept in the context of surveillance remains limited [8, 6]. This is particularly the case in Burkina Faso, where, despite the establishment of an inter-ministerial platform to address health issues, ~~and~~ strong advocacy

and substantial technical and financial support from international organizations and cooperation agencies, zoonosis surveillance remains very poorly integrated and collaborative [9].

Participatory processes were initially proposed in the 1980s as an alternative to more traditional approaches in which dominant stakeholders determine priorities and objectives according to their own interests and agendas [10]. The introduction of participatory processes thus represented an attempt to give a greater voice to categories of stakeholders usually excluded from decision-making [10]. Rowe and Frewer (2004) [11] define participation as "the practice of consulting and involving relevant stakeholders in the identification of priorities, decision-making and policy-making activities of organizations or institutions responsible for policy development". In recent decades, participation has grown significantly and is increasingly mobilized to involve all stakeholders in decision-making to address issues in a variety of fields, such as technology, the environment and health [12].

In this vast field of participation, participatory planning is a particular method that aims to establish a dialogue between various stakeholders to collectively define the actions to be implemented to achieve a common goal [12]. In this approach, it is postulated that engaging beneficiaries early in the planning process establishes a collective vision that can be more effectively realized. The use of a participatory planning process therefore emerged as a contribution to the operationalization of One Health surveillance in Burkina Faso, by offering surveillance stakeholders and beneficiaries a framework for the collective definition of the desired surveillance system, and the means to achieve it.

To this end, a participatory workshop was held with representatives of the different categories of surveillance stakeholders to collectively define such a shared vision, and then identify the changes, and associated actions, necessary to evolve from the current situation to the desired situation.

Materials and methods

To conduct the participatory process, we developed a method inspired by the one proposed by Bordier et al., 2019 [13], which we implemented during a three-day workshop. We will first describe the general organization of the workshop, before detailing the various steps of the method, namely: (i) identification of a shared vision of the One Health surveillance system of anthrax in Burkina Faso; (ii) co-construction of a collective representation of the current situation of the surveillance system in place, in the form of a stakeholder diagram; (iii) identification of the changes needed to move from the current surveillance system to the desired surveillance system; and (iv) drafting of an action plan to achieve the vision of the future One Health surveillance system (Fig 1).

Fig 1. The four steps in the participatory process for the co-construction of the One Health **surveillance system of anthrax in Burkina Faso.**

Workshop organization

In a co-construction process, the quality of the participants is crucial, as their representativeness has a ~~very strong~~ influence on the ~~ability~~ of the process to produce results that represent the vision and expectations of all categories of stakeholders. The workshop participants were selected based on a mapping of anthrax surveillance stakeholders in Burkina Faso conducted in a previous study [9]. The aim was to gather a representative of each of the actors involved in the various surveillance functions, while maintaining a group size that would be manageable within the framework of a participatory process. As a result, 16 institutional representatives were invited to the workshop. Table 1 summarizes the list of invited institutions. “Local authorities” refers to field actors working for ministries in charge of public health, animal health and wildlife conservation. In the case of this workshop, we chose an easily accessible meeting place, outside any sectoral ~~ministry~~, so as not to give more weight to one sector than another. Invitations were issued by the Ministry of Higher Education, Research and Innovation.

Table 1. List of invited institutions and number of participants.

Sector	Category of actors	Number of participants invited	Number of participants present		
			1st day	2nd day	3rd day
Multisectoral	National authorities	4	1	2	2
Animal health	Central authorities	1	1	1	1
	Local authorities	2	2	2	2
	Central laboratory	1	0	0	0
	Regional laboratory	1	1	1	1
Human health	Central authorities	1	1	1	1
	Local authorities	1	1	0	1
Wildlife conservation	Central authorities	1	1	1	1
	Local authorities	2	2	2	2
International aid	International technical and financial partners	2	2	1	1

Facilitation is ~~also~~ an important element in the success of a participatory process; the quality of facilitation determines the relevance of the results produced during the workshop. Facilitators need to be agile in leading discussions with participants from different backgrounds, using appropriate participative tools. Their role includes encouraging participants to clarify what they are saying when there is a risk of misinterpretation, and even to rephrase, if necessary, to ensure that the whole audience understands [14, 15]. The co-construction workshop was facilitated by two researchers (one specializing in epidemiological surveillance and another in complex systems and support modelling) and a PhD student from Burkina Faso working on integrated surveillance of anthrax in the country. The process included an evaluation plan to assess the quality of the results produced, as well as the effects

of the workshop on participants and on the wider implementation context. Qualitative and semi-quantitative indicators were measured based on data collected before, during and after the workshop, using different methods (questionnaires, semi-structured interviews, workshop observation). This plan will not be presented here but will be the subject of another publication.

Identification of a shared vision for a One Health surveillance system for anthrax in Burkina Faso

Before starting the discussions towards a shared vision of the desired surveillance system, the first step was to collectively agree on a definition of "integrated surveillance system" based on a One Health approach for the purposes of the exercise. Each participant was asked to write down a word or a short phrase which, in their opinion, defines or characterizes a One Health integrated surveillance system. The proposals made by the participants were then grouped together by theme to arrive at a common definition of One Health surveillance. The next step was to define a common vision of the One Health surveillance system over a 10-year timeframe. This seemed to be the most appropriate timeframe to enable participants to envision a functional One Health surveillance system in the context of Burkina Faso. Participants were encouraged to write a short narrative about their vision of the ideal system in terms of objectives, performance, organization and operation, and the role they see themselves playing in it. The participants took it in turns to read their notes, and a thematic grouping of the various characteristics of the desired system was drawn up as they went along by the co-facilitator (Photo 1).

Photo 1. Collective definition of the vision of the One Health surveillance system for anthrax desired in 10 years' time in Burkina Faso.

Co-construction of a collective representation of anthrax surveillance in Burkina Faso in the form of a stakeholder diagram

To enable participants to project the changes required to achieve the desired One Health surveillance system, it was necessary for them to begin by collectively defining a common representation of the current organization and operation of surveillance. To this end, they were supported in the co-construction of an actor diagram focusing on information flow, in which the roles and missions of all the actors involved in the surveillance system were represented, as well as their interactions. The aim was for participants to learn from each other and produce new knowledge, fostering the development of mutual understanding [15]. A preliminary actor diagram, drawn up in advance of the workshop based on the work of Nana et al. (2022) [9], was presented to the participants. Participants were invited to propose modifications after pointing out their place in the diagram to the whole audience. The facilitator made modifications as they went, after validating them with all the participants (Photo 2).

Photo 2. Collective revision of the diagram of anthrax surveillance actors in Burkina Faso.

Identification of the changes needed to achieve the desired vision

A "backcasting" method was used to trace backwards from the vision of the future to the current situation. This method is notably used in anticipatory approach to help stakeholders define a roadmap for achieving sustainable and desirable futures at the scale of a given territory and in the context of a specific issue [16].

In this step, workshop participants were asked to identify the changes needed to move from the current situation to their vision of the future. The characteristics of the desired situation were first mentioned at one end of a timeline, then, for each of them, a description of the current situation was collectively defined and mentioned at the other end of the timeline. The facilitator then asked participants to record, by writing on a card, any changes needed to reach each desired characteristics, starting from the future and working backward to the present. Changes were expressed in terms "which actor needs to do what differently". The facilitator asked participants to arrange the changes in chronological relation to each other until a path of changes was constructed, to move from the current situation to

the desired situation (Fig 2). The participants then identified causal links between the various proposed changes.

Fig 2. Change pathway model used to construct the transition from the current to the desired situation of the One Health surveillance system for anthrax in Burkina Faso.

Design of an action plan to operationalize changes to achieve the desired vision

Once the changes needed to achieve the desired vision of the One Health surveillance system for anthrax had been identified, participants drafted an action plan to operationalize the changes identified. This reflective step was also an opportunity to identify levers and obstacles regarding those changes, which were also recorded on the pathway of changes. Once this consolidation work had been completed, the changes were considered one by one and participants asked to identify the necessary actions for their respective implementation, and to characterize them in terms of: beneficiaries; responsible; implementer; source of funding; possible levers and obstacles regarding implementation; date; and duration of implementation. Due to time constraints and the disruption of the workshop by external political events, the action plan could only be partially outlined.

Ethics approval and consent to participation

This study was evaluated and validated by the ethics committee of the Ministry of Higher Education, Scientific Research and Innovation of Burkina Faso in March 2021 by deliberation n° 2021–07-161. A written informed consent to workshop participation was obtained from all participants. The study protocol complies with applicable guidelines. The recruitment period of participants started on 7 August 2022 with the selection of individuals to be invited and ended on 3 April 2023 with the completion of all interviews conducted to assess the effects of the co-construction process (not presented here).

Results

The co-construction workshop took place from 28 to 30 September 2022 in Ouagadougou, Burkina Faso, in a neutral place, outside of any sectoral ~~ministries~~. The number of participants varied throughout the co-construction workshop: 12 on the first and third days, and 11 on the second day. The number of participants on each day by sector and stakeholder category is shown in Table 1.

A shared vision of One Health surveillance for anthrax in Burkina Faso

The discussion on the definition of integrated surveillance based on a One Health approach led to an agreed definition of the term. Workshop participants defined it as a coordinated and collaborative system between different sectors, enabling ~~the~~ sharing of data and information needed for effective risk management. Such a system requires the production of quality data ~~and is~~ based on the principle of transparency between all actors. The participants agreed on a definition of an ~~integrated~~ anthrax surveillance system within 10 years, based on three pillars: (i) notification and investigation of all cases of anthrax in Burkina Faso; (ii) proper circulation and use of the information generated by surveillance; and (iii) effective intersectoral governance of surveillance. These three pillars result from the thematic grouping based on the characteristics of the ideal system proposed by the participants, and are represented in Fig 3.

Fig 3. Characteristics of the One Health surveillance system for anthrax desired in 10 years' time in Burkina Faso.

Collective representation of the anthrax surveillance system in Burkina Faso in the form of an actor diagram

The revision of the actor diagram led to the addition and deletion of actors and interactions. All these changes were made with a high degree of consensus, except for the interactions linked to information sharing between field actors, which gave rise to differences of opinion. In fact, these exchanges are not formalized in an official document, and, as a result, actors from central authorities wanted them not to

be represented. Local actors objected, arguing that these interactions existed and were often more effective in managing health events than those taking place at central level, which are formalized. A compromise was reached to distinguish formalized and non-formalized interactions on the diagram. The diagram thus amended highlights the importance of informal collaboration in surveillance, and is illustrated in Fig 4.

Fig 4. Diagram of the actors in anthrax surveillance system in Burkina Faso.

Changes needed to achieve the desired vision

Changes needed to achieve the notification and investigation of all cases

The proposed changes had four specific objectives (Figs 5a and 5b). The first was to improve the capacity of local authorities to recognise suspected cases of anthrax (through posters, picture boxes and training) and to notify them (through forms and the deployment of electronic reporting tools). The second objective was to raise public awareness of the need to notify suspected cases. A third objective was to provide the resources needed to confirm suspected cases, in particular by setting up an emergency fund. The final objective was to improve notification procedures by deploying electronic reporting tools for community workers.

Fig 5 (a and b). Necessary changes identified by participants to ensure notification and investigation of all cases.

Changes required for proper circulation and use of the information generated

Changes for improving the circulation and use of the information have been proposed in three domains (Fig 6). To ensure that the information system enables early warning and rapid response, interoperable information systems should be developed and deployed across the country. The information must then be fed back to field agents and communities using the same channel as the one used for case

notification. Finally, any contaminated land areas must be properly mapped based on an updated risk assessment and their coordinates communicated to farmers.

Fig 6. Necessary changes identified by participants in the co-construction workshop to ensure proper circulation and use of the information generated (RESUREP: Réseau de surveillance épidémiologique (Epidemiological Surveillance System); ZATE: Zone d'Appui Technique à l'Elevage (Livestock Technical Support Zone).

Changes needed for effective cross-sectoral governance of surveillance

Figure 7 illustrates the various changes and their implications for effective cross-sector governance of epidemiological surveillance. First, intersectoral coordination committees for zoonosis surveillance need to be established at sub-national level and the technical, and interpersonal skills of committee members enhanced. Next, a specific intersectoral strategic plan for surveillance must be formalized, regularly updated and integrated into the global intersectoral strategic plan for zoonoses. This integration would give greater visibility to the actions proposed for anthrax surveillance and improve the chances of obtaining funding for their implementation. Lastly, coordination must be improved between actors in charge of surveillance and those in charge of management, notably through information exchange within the local intersectoral committees for zoonoses surveillance.

Fig 7. Necessary changes identified by participants in the co-construction workshop to ensure effective cross-sector governance of surveillance

Draft action plan for implementation of the desired One Health surveillance system

The action plan could only be completed for the first pillar, namely notification and investigation of all anthrax cases in Burkina Faso (Table 2). For this pillar, 13 changes were selected by the participants for inclusion in the action plan, and 37 actions were proposed to implement these changes. Nineteen actions were aimed at improving the technical capacities of surveillance actors, of which 15 aimed at fostering interaction between actors. Three actions were dedicated to mobilizing the financial

resources needed to implement the entire plan. The participants identified levers for and barriers to the implementation of the actions formulated. The existing national One Health platform represents an appropriate governance framework for implementing the actions identified. However, the lack of political will to allocate more resources for surveillance, as well as political instability and insecurity in large parts of the country, were identified as potential barriers to the implementation of many of the plan's actions.

Table 2: Action plan for the notification of all cases of anthrax in Burkina Faso

CHANGES	ACTION		ACTION BENEFICIARIES	MANAGER	EXECUTANT	ORIGIN OF FUNDS
1. Budget is allocated and available	1.1	Draw up an action plan (development/validation workshops)	Key surveillance actors	TS - OHNP	Zoonoses commission (a core group of 10 people to draft 0)	TS- OHNP/ TFP/ Sectoral
	1.2	Advocacy with the government and TFPs	Key surveillance actors	TS - OHNP	Zoonoses commission	TS- OHNP/ TFP/ Sectoral
	1.3	Develop an annual staffing program	Key surveillance actors	TS - OHNP	TS - OHNP, technical directorates	TS- OHNP/ TFP/ Sectoral
2. Agents, including those in abattoirs, are equipped with tablets	2.1	Buy the tablets	Authorities (animal health and environment) and community workers	TS - OHNP	Zoonoses commission	TS- OHNP/ TFP/ Sectoral
	2.2	Distribute the tablets	Authorities (animal health and	TS - OHNP	Zoonoses commission	TS- OHNP/ TFP/ Sectoral

CHANGES		ACTION		ACTION BENEFICIARIES	MANAGER	EXECUTANT	ORIGIN OF FUNDS
				environment) and community workers			
3	Agents have notification forms (paper or electronic) and are trained in their use	3.1	Develop notification forms (paper and electronic) for slaughterhouses and slaughter areas	Abattoirs, ZATE, Areas for abattoir slaughter, Nurses	Zoonoses Commission	Technical directorates	TS- OHNP/ TFP/ Sectoral
		3.2	Update sector-specific fact sheets in electronic format for animal, human and environmental health for harmonization	Service points of the various ministries	Zoonoses Commission	Technical directorates	TS- OHNP/ TFP/ Sectoral
		3.3	Organize training on the use of tablets and electronic notification forms	Authorities (animal health and environment) and community workers	TS - OHNP	TS - OHNP; technical directorates	TS- OHNP/ TFP/ Sectoral
4.	Posters on case definition are displayed in veterinary posts, health centres and	4.1	Create poster support	Service points of the various ministries	TS - OHNP	Zoonoses commission, with communication support	TS- OHNP/ TFP/ Sectoral
		4.2	Print and distribute posters	Service points of the various ministries	TS - OHNP	Zoonoses commission, with communication support	TS- OHNP/ TFP/ Sectoral

CHANGES		ACTION		ACTION BENEFICIARIES	MANAGER	EXECUTANT	ORIGIN OF FUNDS
	eco-guards' offices.						
5.	Agents are trained in anthrax epidemiology and the recognition of suspected cases	5.1	Identify and train epidemiology trainers	Authorities (animal health and environment) and community workers	TS - OHNP	TS - OHNP; technical directorates	TS- OHNP/ TFP/ Sectoral
		5.2	Provide cross-sectoral training for trainers in epidemiology and the recognition of suspected cases	Regional level	Zoonoses commission	National experts in anthrax and epidemiology	TS- OHNP/ TFP/ Sectoral
		5.3	Provide training for agents, adapted to the sector and administrative levels of the beneficiaries, with a common core	Provincial, local level	Zoonoses commission	Agents trained by national anthrax experts	TS- OHNP/ TFP/ Sectoral
6.	The trackers and eco-guards have image boxes that allow them to recognize cases	6.1	Update the image box or create a new box	Eco-guards, trackers	Environment technical directorate	Head of surveillance activities	TS- OHNP/ TFP/ Sectoral
		6.2	Disseminate image boxes based on existing unusual events to all eco-guards and trackers	Eco-guards, trackers	Environment technical directorate	Head of surveillance activities	TS- OHNP/ TFP/ Sectoral

CHANGES		ACTION		ACTION BENEFICIARIES	MANAGER	EXECUTANT	ORIGIN OF FUNDS
7	Community leaders are trained and involved in surveillance activities	7.1	Identify the community leaders who will be involved	Service points of the various ministries, community leaders	Agents of service points, managers of private human or animal health structures	Agents of services points (CSPS, ZATE, PV, PF), managers of private health structures (clinic, human or animal care practice)	TS- OHNP/ TFP
		7.2	Plan meetings with community leaders	Service points of the various ministries, community leaders	Agents of service points, managers of private human or animal health structures	Agents of services posts (CSPS, ZATE, PV, PF), managers of private health structures (clinic, human or animal care practice)	TS- OHNP/ TFP
		7.3	Conduct leaders' courtesy visit with multi-sector teams	Service points of the various ministries, community leaders	Agents of service points, managers of private human or animal health structures	Agents of services posts (CSPS, ZATE, PV, PF), Managers of private health structures (clinic, human or animal care practice)	TS- OHNP/ TFP
		7.4	Sensitize leaders to anthrax and how to notify with appropriate tools (photos, sketches)	Population, leaders, service points of	Zoonosis and surveillance commissions	Agents of services posts (CSPS, ZATE, PV, PF), managers of private health structures	TS- OHNP/ TFP

CHANGES		ACTION		ACTION BENEFICIARIES	MANAGER	EXECUTANT	ORIGIN OF FUNDS
				various ministries, community leaders		(clinic, human or animal care practice)	
8	People are aware of the risk of anthrax and the importance of notifying suspected cases and are trained to do it	8.1	Train actors on the importance of joint communication	Service points of various ministries, private health care workers	Zoonosis and surveillance commissions	OHNP - Decentralized structures	TS- OHNP/ TFP
		8.2	Harmonizing communication tools	Service points of various ministries, private health care workers	Zoonosis and surveillance commissions	OHNP - Decentralized structures	TS- OHNP/ TFP
		8.3	Develop a joint information and awareness program	Service points of various ministries, private health care workers	Zoonosis and surveillance commissions	OHNP - Decentralized structures	TS- OHNP/ TFP
		8.4	Planning the various training courses	Service points of various ministries, private health care workers	Zoonosis and surveillance commissions	Zoonosis and surveillance commissions	TS- OHNP/ TFP

CHANGES		ACTION		ACTION BENEFICIARIES	MANAGER	EXECUTANT	ORIGIN OF FUNDS
		8.5	Organize joint programmes (TV, radio)	Service points of various ministries, private health care workers	Zoonosis and surveillance commissions	OHNP - Decentralized structures	TS- OHNP/ TFP
		8.6	Organize joint anthrax awareness campaigns by the various sectors	Service points of various ministries, private health care workers	Zoonosis and surveillance commissions	OHNP - Decentralized structures	TS- OHNP/ TFP
9	Butchers are trained to recognise anthrax lesions	9.1	Identify butchers	Butchers, slaughterhouse and slaughter area inspectors, ministry service points	Provincial and regional directorates (in charge of inspections)	Provincial and regional directorates (in charge of inspections)	Sectoral/ TFP
		9.2	Plan training sessions	Butchers, abattoir and slaughter area inspectors, ministry service points	Provincial and regional directorates (in charge of inspections)	Provincial and regional directorates (in charge of inspections)	Sectoral/ TFP

CHANGES		ACTION		ACTION BENEFICIARIES	MANAGER	EXECUTANT	ORIGIN OF FUNDS
10	Agents are assigned and empowered according to their motivation	10.1	Evaluate staffing needs by zone	Human resources directorates of the ministries concerned, key surveillance actors	Regional directorates (environment, animal, and human health)	Regional and human resources directorates	Sectoral/ TFP
		10.2	Feedback evaluation results to stakeholders	Human resources directorates of the ministries concerned, key surveillance actors	Regional directorates (environment, animal, and human health)	Regional and human resources directorates	Sectoral/ TFP
		10.3	Create performance indicators	Human resources directorates of the ministries concerned, key surveillance actors	Regional directorates (environment, animal, and human health)	Regional and human resources directorates	Sectoral/ TFP
11	There is effective leadership in the conduct of surveillance activities	11.1	Create concertation frameworks between stakeholders in the ministries concerned	Key surveillance actors	TS - OHNP	OHNP - Decentralized structures	TS- OHNP/ TFP
		11.2	Draft surveillance regulations based on the OH concept	Ministries concerned	TS - OHNP	Zoonoses and surveillance commissions	TS- OHNP/ TFP

CHANGES		ACTION		ACTION BENEFICIARIES	MANAGER	EXECUTANT	ORIGIN OF FUNDS
12	Actions are implemented to motivate agents	12.1	Draw up a motivation grid	Key surveillance actors	TS - OHNP	Zoonoses and surveillance commissions	TS- OHNP/ TFP
		12.2	Send letters of congratulations and awards to deserving actors	Key surveillance actors	TS - OHNP and central directorates of the ministries concerned	Central and regional directorates of the ministries concerned	Sectoral
13	Existing paper and electronic notices are simplified	13.1	Organize a workshop to update data collection sheets	Service points of various ministries, private health care workers	TS - OHNP and central directorates of the ministries concerned	Zoonoses and surveillance commission	TFP
		13.2	Create an electronic form	Service points of various ministries, private health care workers	Zoonosis and surveillance commissions	Information technology directorates of the ministries concerned	TFP
		13.3	Develop an easy-to-use application for filling in electronic forms	Service points of various ministries, private health care workers	Zoonosis and surveillance commissions	Information technology directorates of the ministries concerned	TFP

CSPS: Centre de Santé et de Promotion Sociale (health and social promotion centre; PF: Poste forestier (forestry post); OHNP: One Health National Platform; SS: sanitary services; TFP: technical and financial partners; TS: technical secretary; PV: Poste vétérinaire (Veterinary post); ZATE: Zone d'Appui Technique à l'Elevage (Livestock Technical Support Zone).

Discussion

The co-construction process implemented in Burkina Faso proved fruitful in supporting stakeholders towards the implementation of a One Health surveillance system for anthrax. It led to collective representation of the current surveillance system and of the desired surveillance system, as well as the definition of changes and actions to move from one to the other. However, because of time constraints, the action plan was not finalised.

In the course of defining One Health surveillance, participants from the animal health and environmental sectors demonstrated a good understanding of what surveillance means in a One Health approach, and of the issues involved. Some participants from the human sector, however, were unfamiliar with the concept, and interpreted integrated surveillance as meaning good coordination and collaboration between actors within the same intra-sectoral surveillance programme, operating at different decision-making levels. The definition finally agreed upon by the participants is consistent with those in the literature for integrated surveillance in a One Health approach [17, 13]. The co-construction process, by providing a framework for participants to compare their perspectives, highlighted several points of divergence but also enabled participants to engage in a learning process towards greater mutual understanding.

The quality of a participatory process significantly impacts the quality of its results [18]. The ability of any participatory process to produce relevant results is based on several elements: (i) the representativeness, knowledge and influence of the participants; (ii) the ability of the method to elicit, compile and synthesize information from different sources to create representations to which participants can reasonably adhere; and (iii) the quality of the facilitation [15]. The results produced by the participatory process must therefore be analysed in the light of the quality of the process itself.

Despite our efforts to bring together a panel of participants who are representative of the key actors in the anthrax surveillance system in Burkina Faso, it was difficult to benefit from the presence of certain actors and/or maintain their commitment throughout the workshop. Moreover, it was not just a question of having all categories of stakeholders represented, but also of the representative sent by the invited institution being relevant to the workshop's objective. This was not the case for the representative of the animal health sector at local level, who had neither a role in nor experience of anthrax surveillance. There is also the question of how representative a participant's point of view is of the institution represented. Indeed, points of view can diverge from one individual to another within the same category of stakeholders, and inviting a single representative from a given institution to this type of process is likely to be insufficient to capture and consider exhaustively the knowledge and expectations of that category [9, 19]. Finally, the ability of the process to generate change outside its arena is strongly linked to the participants' power in influencing decision-making. In our case, where the main objective was to produce an action plan, the question of its implementation arises if the collective decisions taken during the workshop are not taken to a high enough decision-making level [8]. Some categories of stakeholders appeared to be more involved than others in the issue of One Health surveillance. Environmental and animal health participants showed a greater interest than their human health counterparts. There are two possible reasons for this. The first is that One Health surveillance is still viewed in a very anthropocentric way. In this scenario, the workshop thus represented an opportunity for the animal and environmental sectors, which have more limited resources for surveillance than the human sector, to argue in favour of a better distribution of available resources between sectors, and also between levels of intervention [8]. The second is that zoonoses remain a marginal issue for the human health sector, particularly compared with infectious diseases of non-animal origin (such as malaria or HIV) and non-infectious diseases (such as diabetes), which represent a greater health and socio-economic impact [20].But ensuring that stakeholders are properly represented is not enough to guarantee the success of a participatory process. The challenge then lies in ensuring that a plurality of viewpoints is expressed and combined to generate new knowledge and

collective decisions. Managing diversity can be complex, posing implementation difficulties that compromise the achievement of the objectives sought through the process [21]. Furthermore, participatory processes require more commitment and energy from participants than traditional approaches, where participants are more passive [22]. The quality of the methodology adopted and the ability of the facilitation team to implement it are therefore key determinants of the success of a participatory process [15, 18]. The composition of the facilitation team and the quality of facilitation are also critical to the success of such a process, as the facilitator or facilitators hold the keys to the change process [11]. On the one hand, it is important to have people in that role who will moderate in an almost “naïve” way and not bring their own opinion to bear on what participants say. On the other hand, it can be beneficial for facilitators to have some expertise in the field, to enable them to challenge participants on potential inconsistencies or areas that may not have been addressed [15]. Indeed, it is not certain that the mere confrontation of different points of view will lead to an accurate analysis of the situation and needs, let alone to a compromise. The facilitator therefore also has a role to play in sharing and debating objective information [11]. In addition, the facilitator's role is to ensure that the plurality of viewpoints is considered. To do this, the facilitator must be able to manage conflicts and power dynamics that may represent an obstacle to the collective process [23]. For our workshop, the facilitation team was diverse, including contextual, technical, and methodological experts.

Despite all the efforts to develop participatory processes that are inclusive, adapted to the context and adaptive, participatory processes are complex and influenced by social and political factors that are beyond the control of the process initiators [10].

The power relationships between institutions and stakeholder categories outside the participatory forum are likely to be reproduced within the process, and some participants are likely to have a greater influence than others on the decisions taken. There is therefore a risk that the participatory process may actually reinforce power imbalances by presenting its results as the fruit of a collective reflection, while the contributions from actors of different categories may have been unequal and the results may

in fact mainly reflect the concerns of the actors from dominant categories [24]. During our workshop, for example, the hierarchical position and high social recognition of some participants sometimes hampered the free debate of ideas and sharing of perspectives. Moreover, the act alone of expressing their point of view publicly does not guarantee that stakeholders from categories with less power will truly be listened to and heard. Indeed, where there are tensions, free expression by participants can even have the opposite effect, by reinforcing those points of tension and power imbalances [11].

In the same vein, the decisions that will be taken, even in a collective and concerted manner, as part of the participatory process, have no certainty of success because they are dependent on elements external to the process [12]. In the case of our participatory process, most proposed actions are dependent on the availability of a sufficient budget, itself largely dependent on funding granted by technical and financial partners, who may have different priorities from those that guided the action plan. Implementation of the action plan co-constructed with the stakeholders therefore lies not so much in the changes engendered in the surveillance stakeholders as in changes in the rules of the game that determine decision-making and financial flows [11].

Finally, despite the quality of the method and facilitation, in some cases it may be difficult to engage certain individuals who have cultural values and cognitive framework that hinder their participation in this type of co-construction process that is based on listening, sharing, and co-learning [10].

The co-construction process implemented in Burkina Faso took place over a three-day workshop. It would have benefited from being more iterative, through the organization of further workshops during which participants could have reflected on the results produced, to discuss and develop them further [14, 25]. This would also have been an opportunity to usefully involve certain categories of stakeholders not included during this first one (farmers, veterinarians and medical practitioners). Moreover, in the case of anthrax surveillance, the front-line actors are those working at local level, and it would have been interesting to organize workshops with a wider range of local actors by decentralizing them to a sub-national level. In addition, it would be interesting to put a figure on the cost of the actions identified to facilitate the search for funding.

The co-construction process proposed in Burkina Faso to collectively define the modalities for implementing the One Health surveillance system for anthrax appears satisfactory in terms of the results produced and the active participation of those invited. Participants expressed their satisfaction regarding the workshop organization and the results produced, and stated that they would take actions to contribute to the application of the decisions taken. They also considered that they improved their knowledge of the epidemiology of anthrax, as well as of the organization and functioning of anthrax surveillance. However, this evaluation remains highly subjective. Despite the anticipated benefits, participatory processes are still largely controversial. They are often criticized by decision-makers as costly in terms of time and resources [25]. They are often questioned because the results produced are very largely dependent on the quality of facilitation and the representativeness of participants [14]. Additionally, from our operational perspective, the results produced cannot claim to be the most suitable and appropriate for the simple reason that they were co-constructed, since they are representative of only part of the viewpoints and were influenced by the social and political context in which they were expressed [10]. It is therefore necessary to explicitly document the implementation of the process, to assess its intrinsic quality (method used, facilitation, representativeness of participants) and to measure its effects (changes engendered among participants, or on the implementation context) in a robust way, while considering other factors that could also have contributed to the observed effects [13]. Monitoring and evaluating the participatory process is also an opportunity to highlight elements that may impact the quality and effects of the process and therefore redirect the course of the process to take these elements into account [12].

Conclusions

The process of co-constructing a One Health surveillance system for anthrax in Burkina Faso enabled representatives of surveillance stakeholders to collectively define their vision of the ideal One Health surveillance system for anthrax. They were then able to establish the path to be taken from the current

situation to this desired vision of anthrax surveillance. This co-construction process was also an opportunity for the participants to engage in a process of technical and social learning, by exchanging their points of view on the problem and the solutions for dealing with it. This pooling of knowledge, which generates new collective knowledge, creates a conducive environment for making collective decisions and planning future action. The interactions that take place between participants during the process provide the basis for new partnerships and collaboration, conducive to the implementation of more integrated health policies.

The methodological framework we developed and used to conduct the participatory process consists in four key steps: defining the ideal situation or vision of the future; characterizing the current situation; identifying changes to achieve the ideal situation; and drafting an action plan to operationalize the changes. However, the implementation of the method needs to be adaptive and iterative, adapting along the way not only to the knowledge shared by the participants, but also to the posture of the participants in relation to the approach and to each other. The adopted method and the quality of facilitation is therefore a key element in the success of the participatory process.

Each participatory process is unique because it is specific to the context in which it is implemented, the participants mobilized, and the objectives pursued [12]. However, our study proposes a methodological framework that can be used, after adaptation, in other contexts to address different issues.

Acknowledgements

The authors would like to thank all the participants who agreed to take part in the participatory process.

References

1. World Health Organization: Tripartite and UNEP support OHHLEP's definition of "One Health": Joint Tripartite (FAO, OIE, WHO) and UNEP Statement. 2021.

https://www.who.int/news/item/01-12-2021-tripartite-and-unep-support-ohhlep-s definition-of-one-health. Accessed 21 October 2023.

2. Hayman DTS, Adisasmito WB, Almuhairi S, Behravesh CB, Bilivogui P, Bukachi SA, et al. Developing One Health surveillance systems. One Health. 2023;100617.
3. Babo Martins S, Rushton J, Stärk KDC. Economic assessment of zoonoses surveillance in a 'One Health' context: a conceptual framework. Zoonoses Public Health. 2016; 63: 386–95.
4. Aenishaenslin C, Häsler B, Ravel A, Parmley J, Stärk K, Buckeridge D. Evidence needed for antimicrobial resistance surveillance systems. Bull World Health. 2019; 97(4): 283–9.
5. Zinsstag J, Utzinger J, Probst-Hensch N, Shan L, Zhou XN. Towards integrated surveillance-response systems for the prevention of future pandemics. Infect Dis Poverty. 2020; 9(1): 140.
6. Bennani H, Cornelsen L, Stärk KDC, Häsler B. Evaluating Integrated Surveillance for Antimicrobial Use and Resistance in England: A Qualitative Study. Front Vet Sci. 2021; 8: 743857.
7. Streichert LC, Sepe LP, Jokelainen P, Stroud CM, Berezowski J, Del Rio Vilas VJ. Participation in One Health Networks and Involvement in the COVID-19 Pandemic Response: A Global Study. Front Public Health.2022; 10: 830893.
8. Bordier M, Uea-Anuwong T, Binot A, Hendrikx P, Goutard FL. Characteristics of One Health surveillance systems: A systematic literature review. Preventive Veterinary Medicine. 2020; 181: 104560.
9. Nana SD, Caffin JH, Duboz R, Antoine-Moussiaux N, Binot A, Diagbouga PS et al. Towards an integrated surveillance of zoonotic diseases in Burkina Faso: the case of anthrax. BMC Public Health. 2022; 22(1): 1535.

10. Lavigne Delville P, Mathieu M. Donner corps aux ambitions : le diagnostic participatif comme enjeu de pouvoir et comme processus social, in Lavigne Delville P, Sellamna NE, Mathieu M Les enquêtes participatives en débat : ambitions, pratiques et enjeux. Paris : Karthala, 2000. Article in French
11. Rowe G, Frewer LJ. Evaluating public-participation exercises: a research agenda. Science, technology, and human values. 2004; 29(4): 512-556.
12. Hassenforder E. Participatory planning, evaluation, and institutional dynamics in African natural resource management. Australian National Universit. 2015.
13. Bordier M, Delavenne C, Nguyen DTT, Goutard FL, Hendrikx P. One Health Surveillance: A Matrix to Evaluate Multisectoral Collaboration. Front Vet Sci. 2019; 6: 109.
14. Groot A and Maarleveld M. Demystifying facilitation in participatory development. IIED, London. 2000.
15. Etienne M. Companion Modelling. A Participatory Approach to Support Sustainable Development. Versailles: QUAE. 2011
16. Phdungsilp A. Futures studies' backcasting method used for strategic sustainable city planning. Futures. 2011; 43(7): 707-714
17. Stärk KDC, Arroyo Kuribreña M, Dauphin G, Vokaty S, Ward MP, Wieland B, et al. One Health surveillance – More than a buzz word? Preventive Veterinary Medicine. 2015; 120(1): 124
18. Glicken J. Getting stakeholder participation 'right': a discussion of participatory processes and possible pitfalls. Environmental Science and Policy. 2000; 3(6): 305-310.
19. Grünenfelder J and Schurr C. Intersectionality – a challenge for development research and practice? Dev. Pract. 2015; 25 (6): 771–784.
20. World Health Organization: Integrating neglected tropical diseases into global health and development: fourth WHO report on neglected tropical diseases. 2017. https://iris.who.int/bitstream/handle/10665/255011/9789241565448-eng.pdf?sequence=1. Accessed 4 August 2023.

21. Weaver L and Cousins JB. Unpacking the participatory process. Journal of Multidisciplinary evaluation. 2004; 1(1): 19-40.

22. Mansuri G and Rao V. Community-based and-driven development: A critical review. The World Bank Research Observer. 2004 ;19(1): 1-39.

23. Pretty JN, Guijt I, Thompson J, Scoones I. Participatory Learning and Action: A Trainer's Guide. London: The International Institute for Environmental and Development. 1995.

24. Lee K, Brumme ZL. Operationalizing the One Health approach: the global governance challenges. Health Policy and Planning. 2013; 28(7): 778

25. Binot A, Duboz R, Promburom P, Phimpraphai W, Cappelle J, Lajaunie C, et al. A framework to promote collective action within the One Health community of practice: Using participatory modelling to enable interdisciplinary, cross-sectoral, and multi-level integration. One Health. 2015; 1: 44

Fig 3

Click here to access/download;Figure;20231201_Figure 3.tif

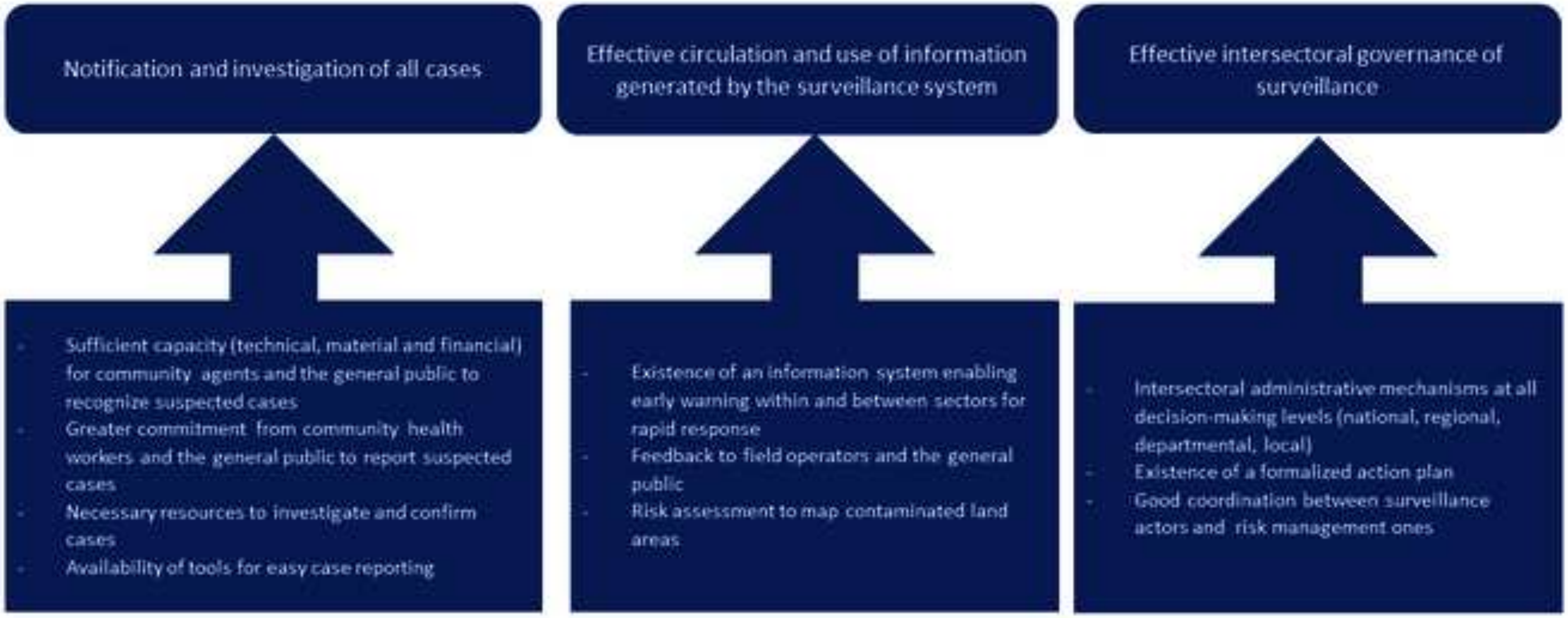

Fig 4

Click here to access/download;Figure;20231213_Figure 4.tif

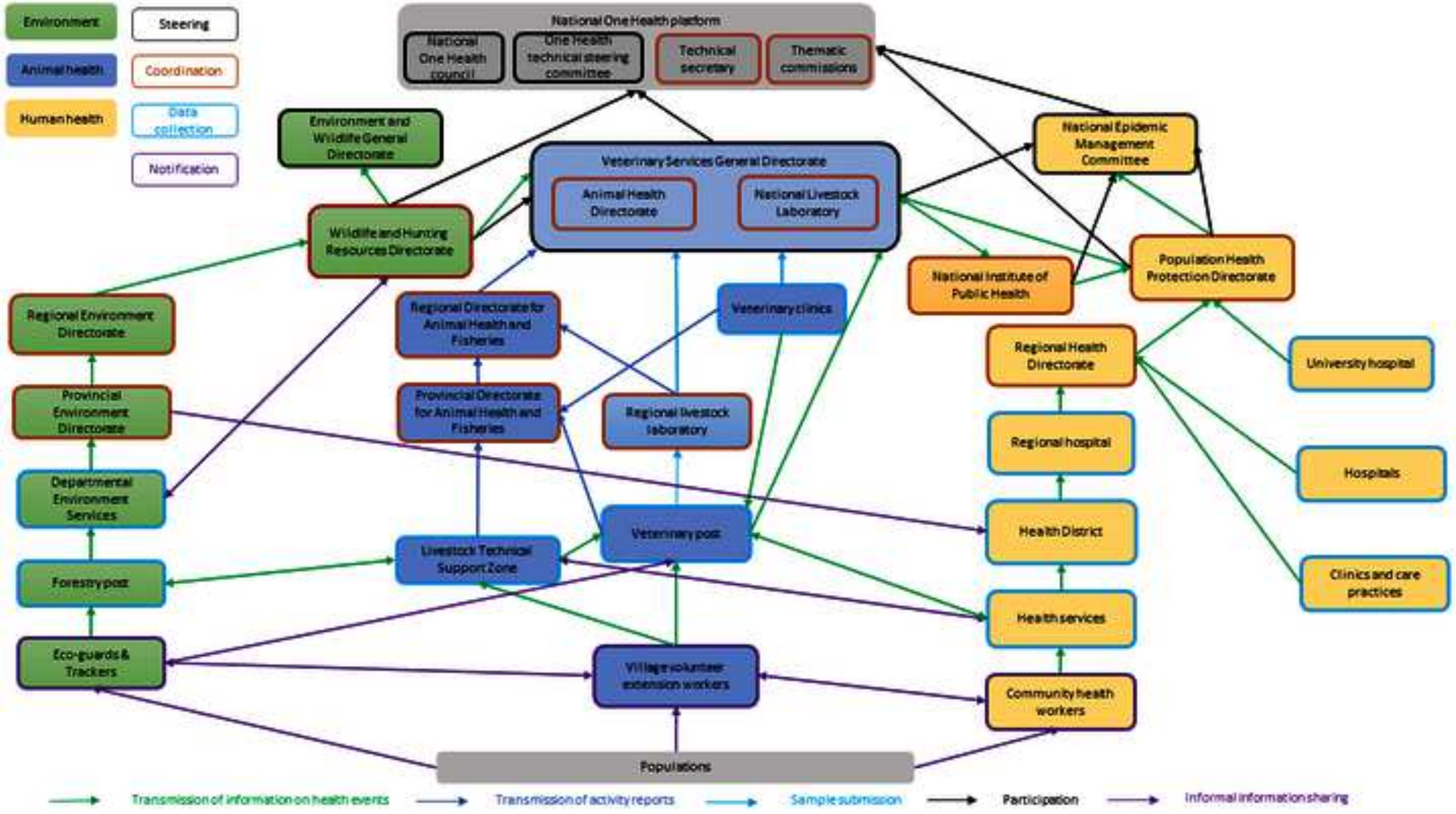

Fig 5

Click here to access/download;Figure;20231213_Figure 5a.tif

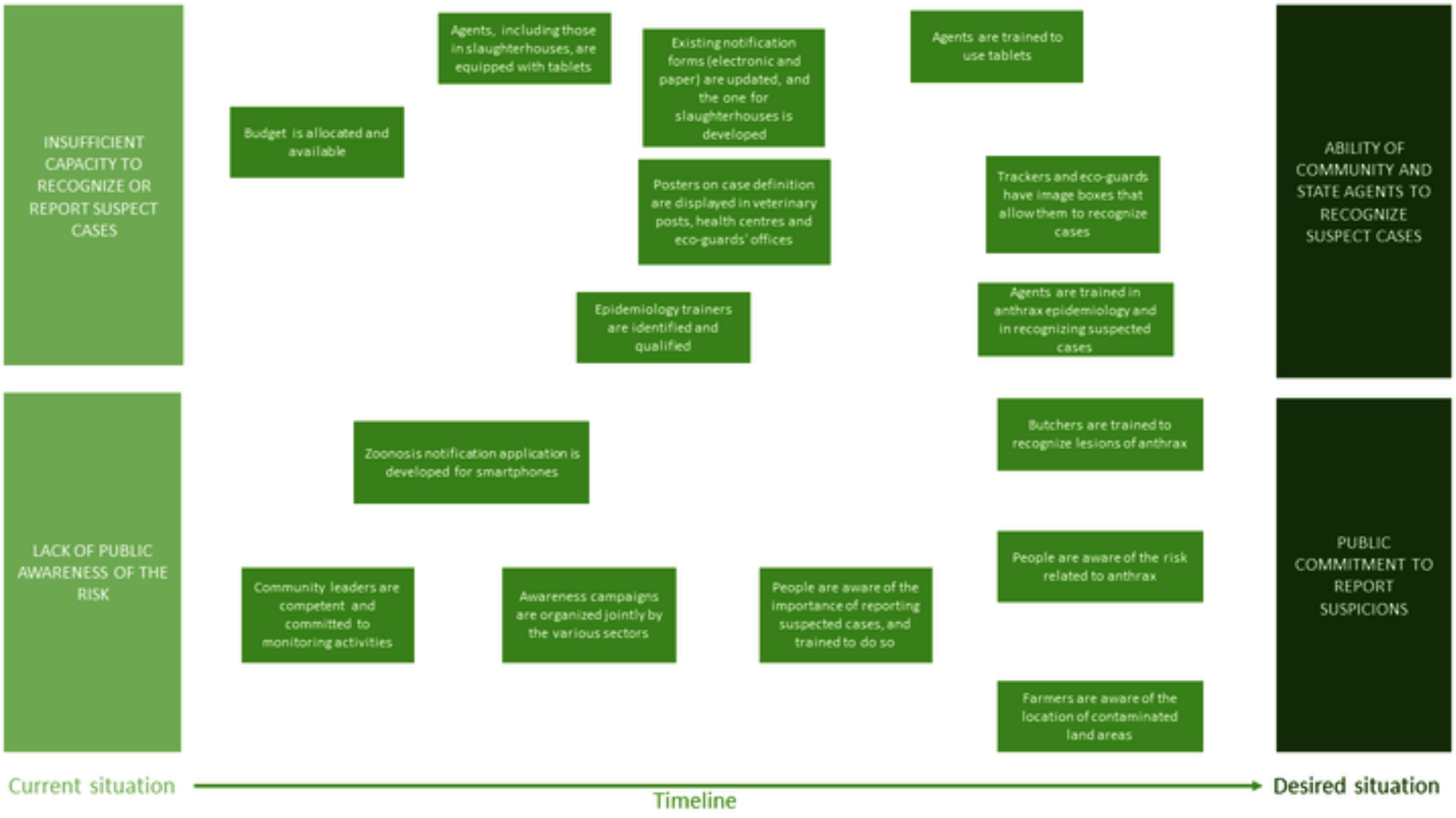

Fig 6 Click here to access/download;Figure;20231213_Figure 5b.tif

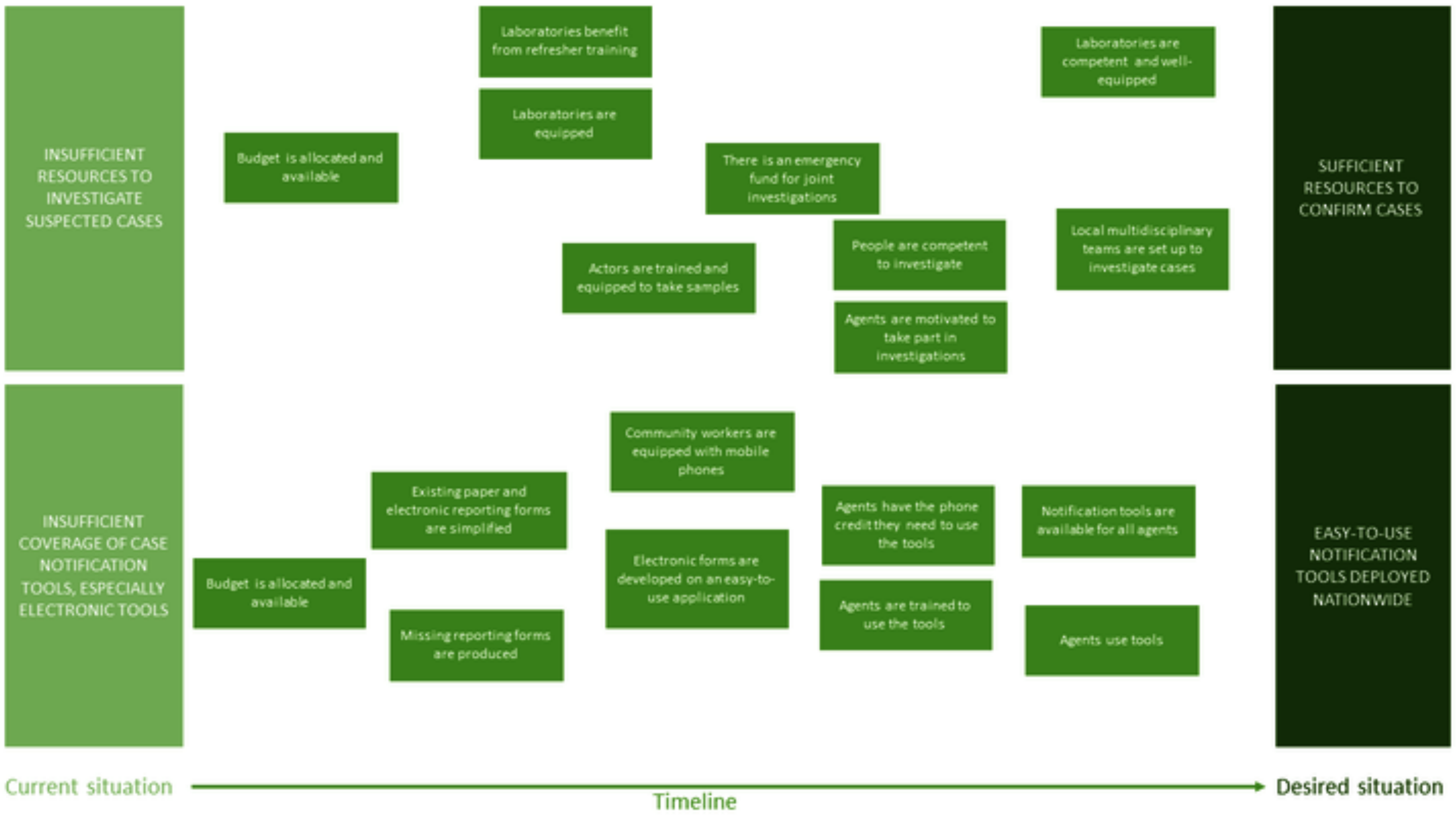

Fig 7

Click here to access/download;Figure;20231213_Figure 6.tif

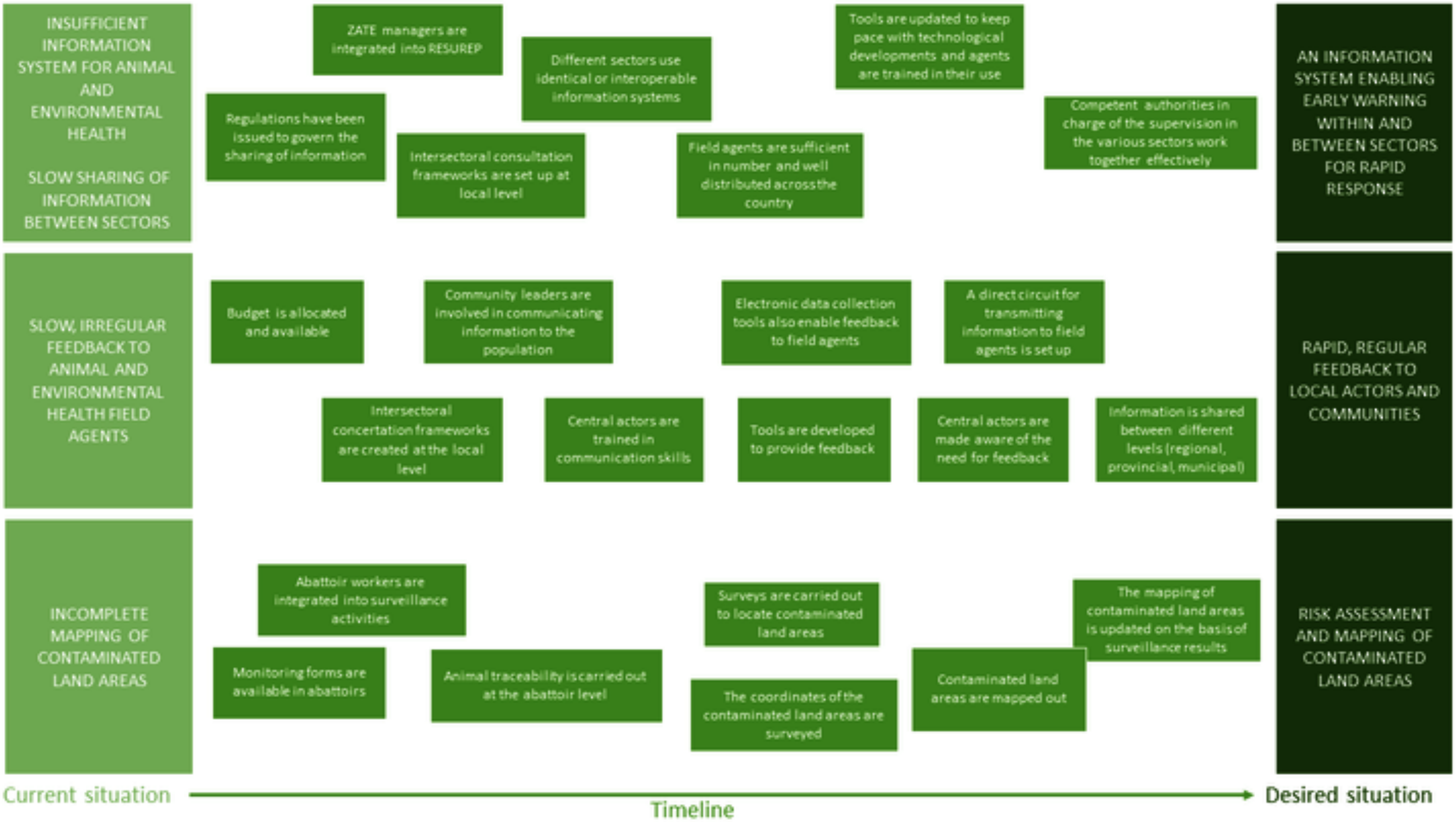

Fig 8

Click here to access/download;Figure;20231213_Figure 7.tif

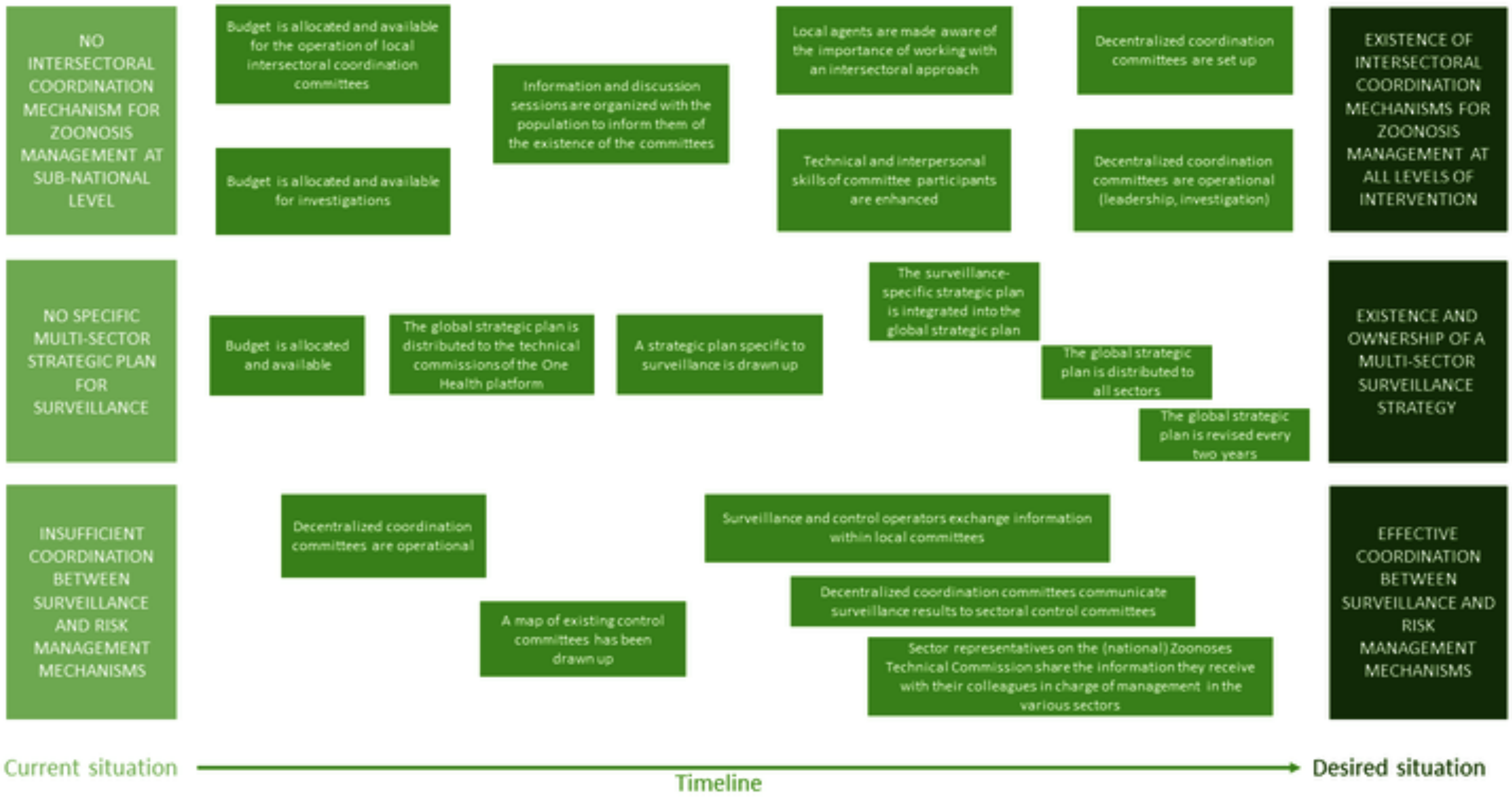

photo 1 Click here to access/download;Figure;20231213_Photo 1.tif

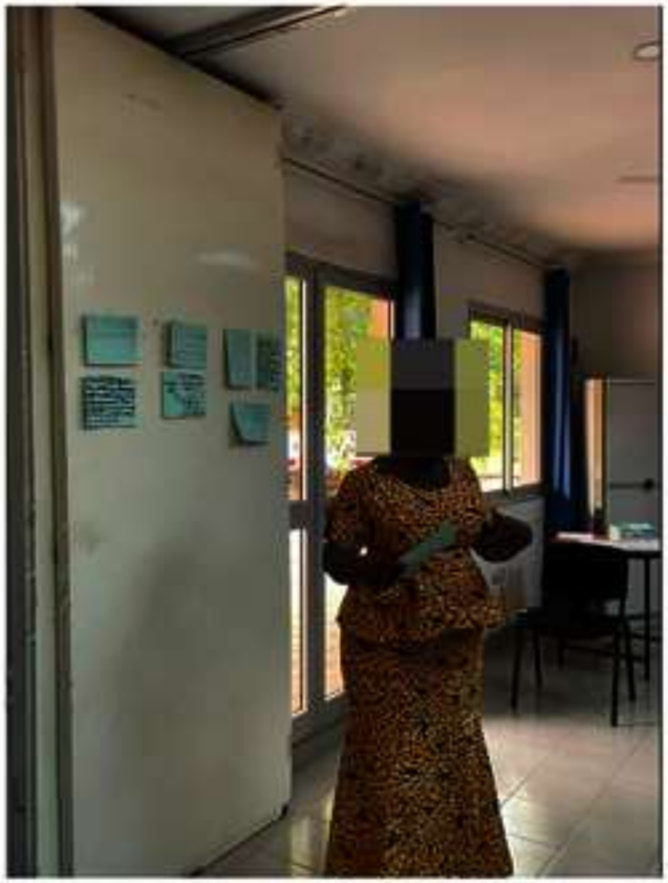

photo 2

Click here to access/download;Figure;20231213_Photo 2.tif

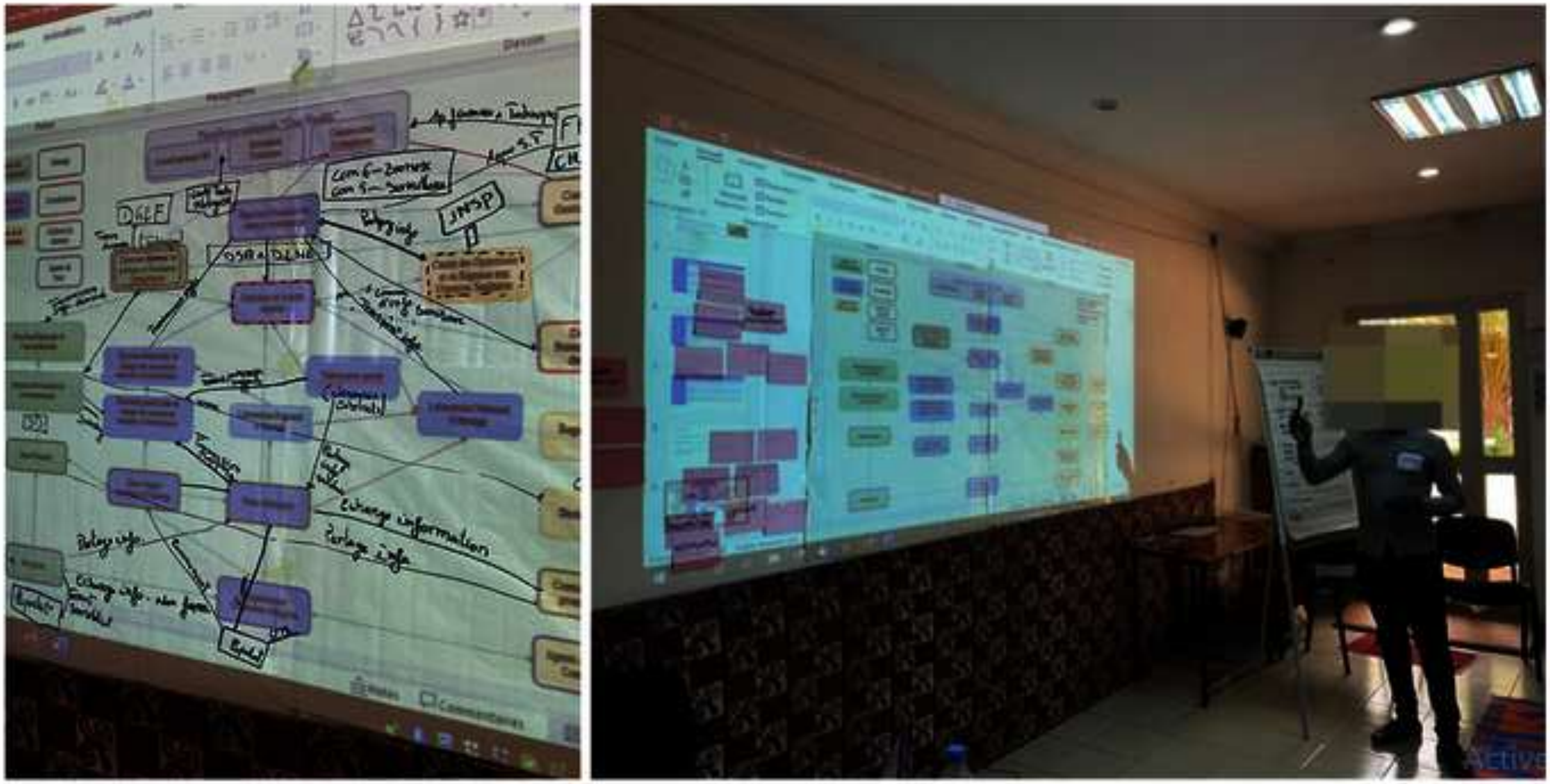

Fig 1

Click here to access/download;Figure;20231201_Figure 1.tif

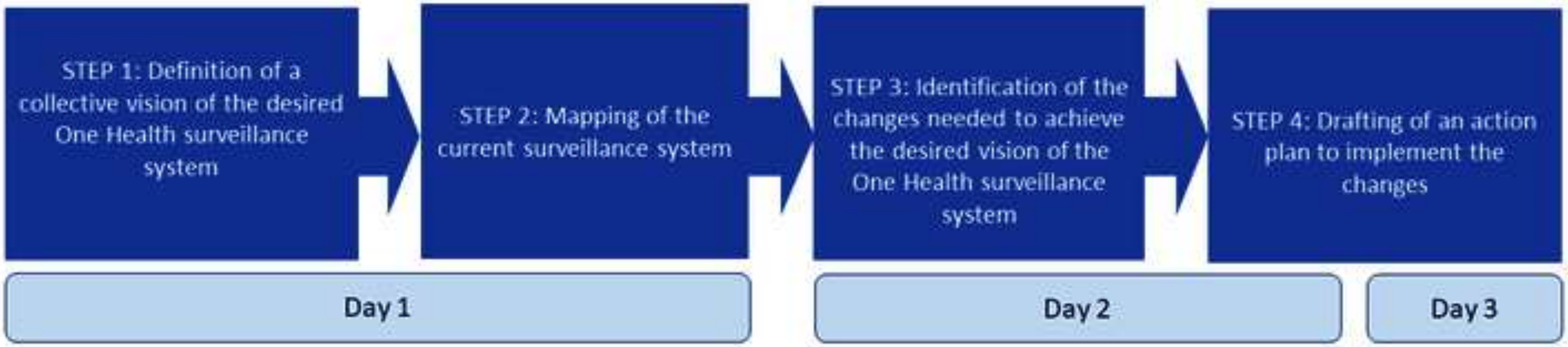

Fig 2

Click here to access/download;Figure;20231201_Figure 2.tif

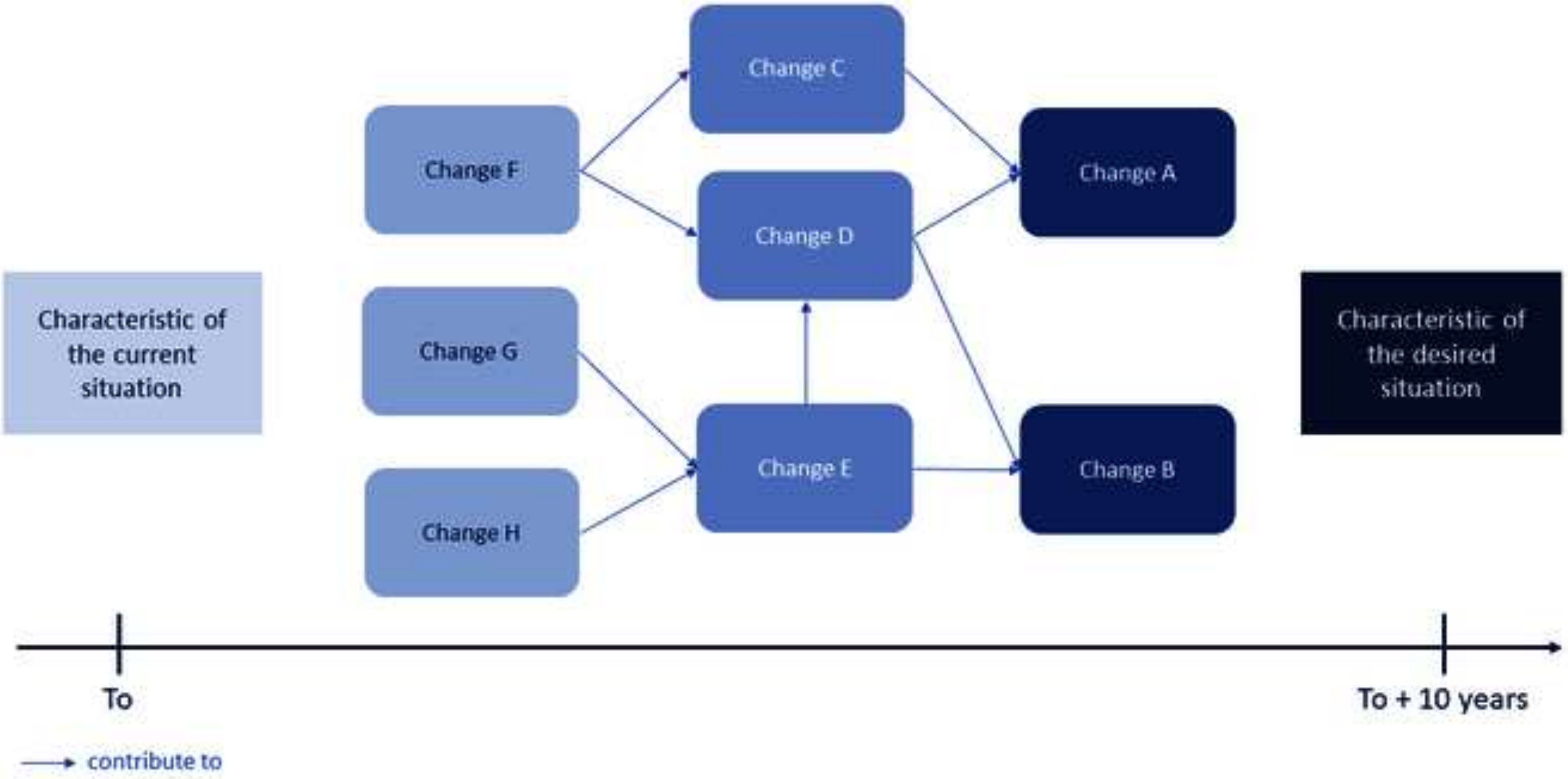

Click here to access/download

Supporting Information

Inclusivity-in-global-research-questionnaire.pdf

A participatory approach to move towards ~~support the development of~~ a One Health surveillance system for anthrax in Burkina Faso

Sougrenoma Désiré Nana [1,2], Raphaël Duboz[1,3,4], Potiandi Serge Diagbouga[5,6], Pascal Hendrikx[7] and Marion Bordier[1,3,8]*

[1] UMR ASTRE, University of Montpellier, CIRAD, INRAE, Montpellier, France

[2] UMR ASTRE, CIRAD, Montpellier, France

[3] UMR ASTRE, CIRAD, Dakar, Senegal

[4] UMMISCO, IRD, Sorbonne University, Bondy, France

[5] Research Institute of Health Sciences, Ouagadougou, Burkina Faso

[6] Health Training and Research &Development, Ouagadougou, Burkina Faso.

[7] High Council for Food, Agriculture and Rural Areas, Paris, France.

[8] National Laboratory for Livestock and Veterinary Research, Senegalese Institute of Research in Agriculture, Dakar, Senegal

*Corresponding author

E-mail: marion.bordier@cirad.fr (MB)

Abstract

The One Health approach calls for collaboration across various sectors and different scales to improve understanding of complex health issues. Regarding epidemiological surveillance, this implies the development of integrated systems that link several surveillance components operating in different domains (human, domestic animals, environment) and involving several actor networks. However, surveillance ~~systems~~ continues to operate in a very compartmentalized way, with little interaction

between sectoral institutions and with the community for the governance and operation of surveillance activities. This is partly explained by the ~~lack of~~insufficient consideration of the local context and the late involvement of ~~, and by only involving~~national stakeholders ~~in the later stages of the~~ when developing programmes that aimed at strengthening the integration of surveillance. In low- and middle-income countries in particular, there is a strong influence of external partners on the development of intersectoral programmes, including surveillance systems. In this context, we developed and implemented a participatory planning process to support stakeholders of the surveillance system of anthrax in Burkina Faso, in the definition of the ~~integrated~~ One Health surveillance system they wish for and of the pathway to reach it. The workshop produced an ~~concrete~~ action plan that reflects the views and perspectives of representatives of the different categories of stakeholders and beneficiaries of surveillance. In addition, the participation of stakeholders in this participatory co-construction process has also improved their knowledge and mutual understanding, fostering a climate of trust conducive to further collaboration for surveillance activities. However, the quality of the participation raises some questions over the results, and contextual factors may have influenced the process. This underlines the need to include a monitoring and evaluation plan in the process to assess its implementation and ability to produce One Health~~integrated~~ surveillance modalities that are appropriate, accepted and applied over the long term.

Introduction

The One Health approach promotes collaboration between sectors, professions, and disciplines to improve the health of humans, ~~domestic~~ animals, and ecosystems on an equitable basis [1]. The application of this concept to epidemiological surveillance is expected to result in greater epidemiological and economic performance of surveillance systems. Indeed, it is expected to improve knowledge of health events and their management while reducing the operational costs of surveillance [2-6]. Less tangible benefits may also occur, such as the establishment of mutual understanding (i.e., understanding one another's thoughts, feelings, and perspectives) and trust between actors, conducive

Formatted: Font: Not Italic

to further and deeper collaboration [4, 7]. However, the operationalization of the principles of the One Health concept in the context of surveillance remains limited [8, 6]. This is particularly the case in Burkina Faso, where, despite the establishment of an inter-ministerial platform to address health issues, and strong advocacy and substantial technical and financial support from international organizations and cooperation agencies, zoonosis surveillance remains very poorly integrated and collaborative [9].

Participatory processes were initially proposed in the 1980s as an alternative to more traditional approaches in which dominant stakeholders determine priorities and objectives according to their own interests and agendas [10]. The introduction of participatory processes thus represented an attempt to give a greater voice to categories of stakeholders usually excluded from decision-making [10]. Rowe and Frewer (2004) [11] define participation as "the practice of consulting and involving relevant stakeholders in the identification of priorities, decision-making and policy-making activities of organizations or institutions responsible for policy development". In recent decades, participation has grown significantly and is increasingly mobilized to involve all stakeholders in decision-making to address issues in a variety of fields, such as technology, the environment and health [12].

In this vast field of participation, participatory planning is a particular method that aims to establish a dialogue between various stakeholders to collectively define the actions to be implemented to achieve a common goal [12]. In this approach, it is postulated that engaging beneficiaries early in the planning process establishes a collective vision that can be more effectively realized. The use of a participatory planning process therefore emerged as a contribution to the operationalization of One Health surveillance in Burkina Faso, by offering surveillance stakeholders and beneficiaries a framework for the collective definition of the desired surveillance system, and the means to achieve it.

To this end, a participatory workshop was held with representatives of the different categories of surveillance stakeholders to collectively define such a shared vision, and then identify the changes, and associated actions, necessary to evolve from the current situation to the desired situation.

Materials and methods

To conduct the participatory process, we developed a method inspired by the one proposed by Bordier et al., 2019 [13], which we implemented during a three-day workshop. We will first describe the general organization of the workshop, before detailing the various steps of the method, namely: (i) identification of a shared vision of the One Healthintegrated surveillance system of anthrax in Burkina Faso; (ii) co-construction of a collective representation of the current situation of the surveillance system in place, in the form of a stakeholder diagram; (iii) identification of the changes needed to move from the current surveillance system achieve to the desired visionsurveillance system; and (iv) drafting of an action plan to achieve the vision of the future One Healthintegrated surveillance system (Fig 1).

Fig 1. The four steps in the participatory process for the co-construction of the One Healthintegrated surveillance system of anthrax in Burkina Faso.

Workshop organization

In a co-construction process, the quality of the participants is crucial, as their representativeness has a very strong influence on the ability of the process to produce results that represent the vision and expectations of all categories of stakeholders. The workshop participants were selected based on a mapping of anthrax surveillance stakeholders in Burkina Faso conducted in a previous study [9]. The aim was to gather a representative of each of the actors involved in the various surveillance functions, while maintaining a group size that would be manageable within the framework of a participatory process. As a result, 16 institutional representatives were invited to the workshop. Table 1 summarizes the list of invited institutions. "Local authorities" refers to field actors working for ministries in charge

of public health, animal health and wildlife conservation. In the case of this workshop, we chose an easily accessible meeting place, outside any sectoral ministry, so as not to give more weight to one sector than another. Invitations were issued by the Ministry of Higher Education, Research and Innovation.

Table 1. List of invited institutions and number of participants.

Sector	Category of actors	Number of participants invited	Number of participants present		
			1st day	2nd day	3rd day
Multisectoral	National authorities	4	1	2	2
Animal health	Central authorities	1	1	1	1
	Local authorities	2	2	2	2
	Central laboratory	1	0	0	0
	Regional laboratory	1	1	1	1
Human health	Central authorities	1	1	1	1
	Local authorities	1	1	0	1
Wildlife conservation	Central authorities	1	1	1	1
	Local authorities	2	2	2	2
International aid	International technical and financial partners	2	2	1	1

Facilitation is also an important element in the success of a participatory process; the quality of facilitation determines the relevance of the results produced during the workshop. Facilitators need to be agile in leading discussions with participants from different backgrounds, using appropriate

participative tools. Their role includes encouraging participants to clarify what they are saying when there is a risk of misinterpretation, and even to rephrase, if necessary, to ensure that the whole audience understands [14, 15]. The co-construction workshop was facilitated by two researchers (one specializing in epidemiological surveillance and another in complex systems and support modelling) and a PhD student from Burkina Faso working on integrated surveillance of anthrax in the country. The process included an evaluation plan to assess the quality of the results produced, as well as the effects of the workshop on participants and on the wider implementation context. Qualitative and semi-quantitative indicators were measured based on data collected before, during and after the workshop, using different methods (questionnaires, semi-structured interviews, workshop observation). This plan will not be presented here but will be the subject of another publication.

Identification of a shared vision for a One Health ~~n integrated anthrax~~ surveillance system for anthrax in Burkina Faso

Before starting the discussions towards a shared vision of the desired surveillance system, the first step was to collectively agree on a definition of "integrated surveillance system" based on a One Health approach for the purposes of the exercise. Each participant was asked to write down a word or a short phrase which, in their opinion, defines or characterizes a One Health~~n~~ integrated surveillance system. The proposals made by the participants were then grouped together by theme to arrive at a common definition of ~~integrated~~ One Health surveillance. The next step was to define a common vision of the One Health~~integrated~~ surveillance system over a 10-year timeframe. This seemed to be the most appropriate timeframe to enable participants to envision a functional One Health surveillance system in the context of Burkina Faso. Participants were encouraged to write a short narrative about their vision of the ideal system in terms of objectives, performance, organization and operation, and the role they see themselves playing in it. The participants took it in turns to read their notes, and a thematic grouping of the various characteristics of the desired system was drawn up as they went along by the co-facilitator (Photo 1).

Photo 1. Collective definition of the vision of the One Health~~**integrated anthrax**~~ **surveillance system** **for anthrax** **desired in 10 years' time in Burkina Faso.**

Co-construction of a collective representation of anthrax surveillance in Burkina Faso in the form of a stakeholder diagram

To enable participants to project the changes required to achieve the desired ~~integrated~~ One Health surveillance system, it was necessary for them to begin by collectively defining a common representation of the current organization and operation of surveillance. To this end, they were supported in the co-construction of an actor diagram focusing on information flow, in which the roles and missions of all the actors involved in the surveillance system were represented, as well as their interactions. The aim was for participants to learn from each other and produce new knowledge, fostering the development of mutual understanding [15]. A preliminary actor diagram, drawn up in advance of the workshop based on the work of Nana et al. (2022) [9], was presented to the participants. Participants were invited to propose modifications after pointing out their place in the diagram to the whole audience. The facilitator made modifications as they went, after validating them with all the participants (Photo 2).

Photo 2. Collective revision of the diagram of anthrax surveillance actors in Burkina Faso.

Identification of the changes needed to achieve the desired vision

A “backcasting” method was used to trace backwards from the vision of the future to the current situation. This method is notably used in anticipatory approach to help stakeholders define a roadmap for achieving sustainable and desirable futures at the scale of a given territory and in the context of a specific issue [16].

In this step, workshop participants were asked to identify the changes needed to move from the current situation to their vision of the future. The characteristics of the desired situation were first mentioned

at one end of a timeline, then, for each of them, a description of the current situation was collectively defined and mentioned at the other end of the timeline. The facilitator then asked participants to record, by writing on a card, any changes needed to reach each desired characteristics, starting from the future and working backward to the present. Changes were expressed in terms "which actor needs to do what differently". The facilitator asked participants to arrange the changes in chronological relation to each other until a path of changes was constructed, to move from the current situation to the desired situation (Fig 2). The participants then identified causal links between the various proposed changes.

Fig 2. Change pathway model used to construct the transition from the current to the desired situation of the One Health **~~integrated anthrax~~ surveillance system for anthrax in Burkina Faso.**

Design of an action plan to operationalize changes to achieve the desired vision

Once the changes needed to achieve the desired vision of the One Health~~integrated~~ surveillance system ~~of~~ for anthrax had been identified, participants drafted an action plan to operationalize the changes identified. This reflective step was also an opportunity to identify levers and obstacles regarding those changes, which were also recorded on the pathway of changes. Once this consolidation work had been completed, the changes were considered one by one and participants asked to identify the necessary actions for their respective implementation, and to characterize them in terms of: beneficiaries; responsible; implementer; source of funding; possible levers and obstacles regarding implementation; date; and duration of implementation. Due to time constraints and the disruption of the workshop by external political events, the action plan could only be partially outlined.

Ethics approval and consent to participation

This study was evaluated and validated by the ethics committee of the Ministry of Higher Education, Scientific Research and Innovation of Burkina Faso in March 2021 by deliberation n° 2021–07-161. A written informed consent to workshop participation was obtained from all participants. The study

protocol complies with applicable guidelines. The recruitment period of participants started on 7 August 2022 with the selection of individuals to be invited and ended on 3 April 2023 with the completion of all interviews conducted to assess the effects of the co-construction process (not presented here).

Results

The co-construction workshop took place from 28 to 30 September 2022 in Ouagadougou, Burkina Faso, in a neutral place, outside of any sectoral ministries. The number of participants varied throughout the co-construction workshop: 12 on the first and third days, and 11 on the second day. The number of participants on each day by sector and stakeholder category is shown in Table 1.

A shared vision of One Health~~integrated~~ ~~anthrax~~ surveillance for anthrax in Burkina Faso

Formatted: Font: 16 pt, Bold

The discussion on the definition of integrated surveillance based on a One Health approach led to an agreed definition of the term. Workshop participants defined it as a coordinated and collaborative system between different sectors, enabling the sharing of data and information needed for effective risk management. Such a system requires the production of quality data and is based on the principle of transparency between all actors. The participants agreed on a definition of an integrated anthrax surveillance system within 10 years, based on three pillars: (i) notification and investigation of all cases of anthrax in Burkina Faso; (ii) proper circulation and use of the information generated by surveillance; and (iii) effective intersectoral governance of surveillance. These three pillars result from the thematic grouping based on the characteristics of the ideal system proposed by the participants, and are represented in Fig 3.

Fig 3. Characteristics of the One Health ~~integrated anthrax~~ **surveillance system for anthrax desired in 10 years' time in Burkina Faso.**

Collective representation of the anthrax surveillance system in Burkina Faso in the form of an actor diagram

The revision of the actor diagram led to the addition and deletion of actors and interactions. All these changes were made with a high degree of consensus, except for the interactions linked to information sharing between field actors, which gave rise to differences of opinion. In fact, these exchanges are not formalized in an official document, and, as a result, actors from central authorities wanted them not to be represented. Local actors objected, arguing that these interactions existed and were often more effective in managing health events than those taking place at central level, which are formalized. A compromise was reached to distinguish formalized and non-formalized interactions on the diagram. The diagram thus amended highlights the importance of informal collaboration in surveillance, and is illustrated in Fig 4.

Fig 4. Diagram of the actors in anthrax surveillance system in Burkina Faso.

Changes needed to achieve the desired vision

Changes needed to achieve the notification and investigation of all cases

The proposed changes had four specific objectives (Figs 5a and 5b). The first was to improve the capacity of local authorities to recognise suspected cases of anthrax (through posters, picture boxes and training) and to ~~report~~ notify them (through forms and the deployment of electronic reporting tools). The second objective was to raise public awareness of the need to ~~report~~ notify suspected cases. A third objective was to provide the resources needed to confirm suspected cases, in particular by setting up an emergency fund. The final objective was to improve ~~reporting~~ notification procedures by deploying electronic reporting tools for community workers.

Fig 5 (a and b). Necessary changes identified by participants to ensure notification and investigation of all cases.

Changes required for proper circulation and use of the information generated

Changes for improving the circulation and use of the information have been proposed in three domains (Fig 6). To ensure that the information system enables early warning and rapid response, interoperable information systems should be developed and deployed across the country. The information must then be fed back to field agents and communities using the same channel as the one used for case notification. Finally, any contaminated land areas must be properly mapped based on an updated risk assessment and their coordinates communicated to farmers.

Fig 6. Necessary changes identified by participants in the co-construction workshop to ensure proper circulation and use of the information generated (RESUREP: Réseau de surveillance épidémiologique (Epidemiological Surveillance System); ZATE: Zone d'Appui Technique à l'Elevage (Livestock Technical Support Zone).

Changes needed for effective cross-sectoral governance of surveillance

Figure 7 illustrates the various changes and their implications for effective cross-sector governance of epidemiological surveillance. First, intersectoral coordination committees for zoonosis surveillance need to be established at sub-national level and the technical, and interpersonal skills of committee members enhanced. Next, a specific intersectoral strategic plan for surveillance must be formalized, regularly updated and integrated into the global intersectoral strategic plan for zoonoses. This integration would give greater visibility to the actions proposed for anthrax surveillance and improve the chances of obtaining funding for their implementation. Lastly, coordination must be improved between actors in charge of surveillance and those in charge of management, notably through information exchange within the local intersectoral committees for zoonoses surveillance.

Fig 7. Necessary changes identified by participants in the co-construction workshop to ensure effective cross-sector governance of surveillance

Draft action plan for implementation of the desired One Health~~integrated~~ surveillance system

Formatted: Font: 14 pt, Bold

The action plan could only be completed for the first pillar, namely notification and investigation of all anthrax cases in Burkina Faso (Table 2). For this pillar, 13 changes were selected by the participants for inclusion in the action plan, and 37 actions were proposed to implement these changes. Nineteen actions were aimed at improving the technical capacities of surveillance actors, of which 15 aimed at fostering interaction between actors. Three actions were dedicated to mobilizing the financial resources needed to implement the entire plan. The participants identified levers for and barriers to the implementation of the actions formulated. The existing national One Health platform represents an appropriate governance framework for implementing the actions identified. However, the lack of political will to allocate more resources for~~improve~~ surveillance, as well as political instability and insecurity in large parts of the country, were identified as potential barriers to the implementation of many of the plan's actions.

Table 2: Action plan for the notification of all cases of anthrax in Burkina Faso

CHANGES		ACTION	ACTION BENEFICIARIES	MANAGER	EXECUTANT	ORIGIN OF FUNDS
1. Budget is allocated and available	1.1	Draw up an action plan (development/validation workshops)	Key surveillance actors	TS - OHNP	Zoonoses commission (a core group of 10 people to draft 0)	TS- OHNP/ TFP/ Sectoral
	1.2	Advocacy with ~~TFPs and~~ the government and TFPs	Key surveillance actors	TS - OHNP	Zoonoses commission	TS- OHNP/ TFP/ Sectoral
	1.3	Develop an annual staffing program	Key surveillance actors	TS - OHNP	TS - OHNP, technical directorates	TS- OHNP/ TFP/ Sectoral
2. Agents, including those in abattoirs, are equipped with tablets	2.1	Buy the tablets	Authorities (animal health and environment) and community workers	TS - OHNP	Zoonoses commission	TS- OHNP/ TFP/ Sectoral
	2.2	Distribute the tablets	Authorities (animal health and	TS - OHNP	Zoonoses commission	TS- OHNP/ TFP/ Sectoral

Formatted Table

CHANGES		ACTION		ACTION BENEFICIARIES	MANAGER	EXECUTANT	ORIGIN OF FUNDS
				environment) and community workers			
3	Agents have notification forms (paper or electronic) and are trained in their use	3.1	Develop notification forms (paper and electronic) for slaughterhouses and slaughter areas	Abattoirs, ZATE, Areas for abattoir slaughter, Nurses	Zoonoses Commission	Technical directorates	TS- OHNP/ TFP/ Sectoral
		3.2	Update sector-specific fact sheets in electronic format for animal, human and environmental health for harmonization	Service points of the various ministries	Zoonoses Commission	Technical directorates	TS- OHNP/ TFP/ Sectoral
		3.3	Organize training on the use of tablets and electronic notification forms	Authorities (animal health and environment) and community workers	TS - OHNP	TS - OHNP; technical directorates	TS- OHNP/ TFP/ Sectoral
4.	Posters on case definition are displayed in veterinary posts, health centres and	4.1	Create poster support	Service points of the various ministries	TS - OHNP	Zoonoses commission, with communication support	TS- OHNP/ TFP/ Sectoral
		4.2	Print and distribute posters	Service points of the various ministries	TS - OHNP	Zoonoses commission, with communication support	TS- OHNP/ TFP/ Sectoral

Formatted Table

	CHANGES		ACTION	ACTION BENEFICIARIES	MANAGER	EXECUTANT	ORIGIN OF FUNDS
	eco-guards' offices.						
5.	Agents are trained in anthrax epidemiology and the recognition of suspected cases	5.1	Identify and train epidemiology trainers	Authorities (animal health and environment) and community workers	TS - OHNP	TS - OHNP; technical directorates	TS- OHNP/ TFP/ Sectoral
		5.2	Provide cross-sectoral training for trainers in epidemiology and the recognition of suspected cases	Regional level	Zoonoses commission	National experts in anthrax and epidemiology	TS- OHNP/ TFP/ Sectoral
		5.3	Provide training for agents, adapted to the sector and administrative levels of the beneficiaries, with a common core.	Provincial, local level	Zoonoses commission	Agents trained by national anthrax experts	TS- OHNP/ TFP/ Sectoral
6.	The trackers and eco-guards have image boxes that allow them to recognize cases	6.1	Update the image box or create a new box	Eco-guards, trackers	Environment technical directorate	Head of surveillance activities	TS- OHNP/ TFP/ Sectoral
		6.2	Disseminate image boxes based on existing unusual events to all eco-guards and trackers	Eco-guards, trackers	Environment technical directorate	Head of surveillance activities	TS- OHNP/ TFP/ Sectoral

Formatted Table

CHANGES		ACTION		ACTION BENEFICIARIES	MANAGER	EXECUTANT	ORIGIN OF FUNDS
7	Community leaders are trained and involved in surveillance activities	7.1	Identify the community leaders who will be involved	Service points of the various ministries, community leaders	Agents of service points, managers of private human or animal health structures	Agents of services points (CSPS, ZATE, ~~VP~~PV, ~~FP~~PF), managers of private health structures (clinic, human or animal care practice)	TS- OHNP/ TFP
		7.2	Plan meetings with community leaders	Service points of the various ministries, community leaders	Agents of service points, managers of private human or animal health structures	Agents of services posts (CSPS, ZATE, PV, PF~~VP, FP~~), managers of private health structures (clinic, human or animal care practice)	TS- OHNP/ TFP
		7.3	Conduct ~~L~~leaders' courtesy visit with multi-sector teams	Service points of the various ministries, community leaders	Agents of service points, managers of private human or animal health structures	Agents of services posts (CSPS, ZATE, PV, PF~~VP, FP~~), Managers of private health structures (clinic, human or animal care practice)	TS- OHNP/ TFP
		7.4	Sensitize leaders to anthrax and how to notify with appropriate tools (photos, sketches)	Population, leaders, service points of various ministries ~~(FP,~~	Zoonosis and surveillance commissions	Agents of services posts (CSPS, ZATE, PV, PF~~VP, FP~~), managers of private health structures	TS- OHNP/ TFP

Formatted Table

	CHANGES		ACTION	ACTION BENEFICIARIES	MANAGER	EXECUTANT	ORIGIN OF FUNDS
				~~SS, VP)~~, community leaders		(clinic, human or animal care practice)	
8	People are aware of the risk of anthrax and the importance of ~~reporting~~ notifying suspected cases and are trained to do it	8.1	Train actors on the importance of joint communication	Service points of various~~different~~ ministries, private health care workers	Zoonosis and surveillance commissions	OHNP - Decentralized structures	TS- OHNP/ TFP
		8.2	Harmonizing communication tools	Service points of various~~different~~ ministries, private health care workers	Zoonosis and surveillance commissions	OHNP - Decentralized structures	TS- OHNP/ TFP
		8.3	Develop a joint information and awareness program	Service points of various~~different~~ ministries, private health care workers	Zoonosis and surveillance commissions	OHNP - Decentralized structures	TS- OHNP/ TFP
		8.4	Planning the various training courses	Service points of various~~different~~ ministries, private health care workers	Zoonosis and surveillance commissions	Zoonosis and surveillance commissions	TS- OHNP/ TFP

Formatted Table

CHANGES		ACTION		ACTION BENEFICIARIES	MANAGER	EXECUTANT	ORIGIN OF FUNDS
		8.5	Organize joint programmes (TV, radio)	Service points of various ~~different~~ ministries, private health care workers	Zoonosis and surveillance commissions	OHNP - Decentralized structures	TS- OHNP/ TFP
		8.6	Organize joint anthrax awareness campaigns by the various sectors	Service points of various ~~different~~ ministries, private health care workers	Zoonosis and surveillance commissions	OHNP - Decentralized structures	TS- OHNP/ TFP
9	Butchers are trained to recognise anthrax lesions	9.1	Identify butchers	Butchers, slaughterhouse and slaughter area inspectors, ministry service points	Provincial and regional directorates (in charge of inspections)	Provincial and regional directorates (in charge of inspections)	Sectoral/ TFP
		9.2	Plan~~ning~~ training sessions	Butchers, abattoir and slaughter area inspectors, ministry service points	Provincial and regional directorates (in charge of inspections)	Provincial and regional directorates (in charge of inspections)	Sectoral/ TFP

Formatted Table

CHANGES		ACTION		ACTION BENEFICIARIES	MANAGER	EXECUTANT	ORIGIN OF FUNDS
10	Agents are assigned and empowered according to their motivation	10.1	Evaluate staffing needs by zone	Human resources directorates of the ministries concerned, key surveillance actors	Regional directorates (environment, animal, and human health)	Regional and human resources directorates	Sectoral/ TFP
		10.2	Feedback evaluation results to stakeholders	Human resources directorates of the ministries concerned, key surveillance actors	Regional directorates (environment, animal, and human health)	Regional and human resources directorates	Sectoral/ TFP
		10.3	Create performance indicators	Human resources directorates of the ministries concerned, key surveillance actors	Regional directorates (environment, animal, and human health)	Regional and human resources directorates	Sectoral/ TFP
11	There is effective leadership in the conduct of surveillance ~~ES~~ activities	11.1	Create concertation frameworks between stakeholders in the ministries concerned	Key surveillance actors	TS - OHNP	OHNP - Decentralized structures	TS- OHNP/ TFP
		11.2	Draft ~~ES~~ surveillance regulations based on the OH concept	Ministries concerned	TS - OHNP	Zoonoses and surveillance commissions	TS- OHNP/ TFP

Formatted Table

CHANGES		ACTION		ACTION BENEFICIARIES	MANAGER	EXECUTANT	ORIGIN OF FUNDS
12	Actions are implemented to motivate agents	12.1	Draw~~ing~~ up a motivation grid	Key surveillance actors	TS - OHNP	Zoonoses and surveillance commissions	TS- OHNP/ TFP
		12.2	Send letters of congratulations and awards to deserving actors	Key surveillance actors	TS - OHNP and central directorates of the ministries concerned	Central and regional directorates of the ministries concerned	Sectoral
13	Existing paper and electronic notices are simplified	13.1	Organize a workshop to update data collection sheets	Service points of various~~different~~ ministries, private health care workers	TS - OHNP and central directorates of the ministries concerned	Zoonoses and surveillance commission	TFP
		13.2	Create an electronic form	Service points of various~~different~~ ministries, private health care workers	Zoonosis and surveillance commissions	Information technology directorates of the ministries concerned	TFP
		13.3	Develop an easy-to-use application for filling in electronic forms	Service points of various~~different~~ ministries, private health care workers	Zoonosis and surveillance commissions	Information technology directorates of the ministries concerned	TFP

CSPS: Centre de Santé et de Promotion Sociale (~~Health~~ health and ~~Social~~ social p~~P~~romotion c~~C~~entre; ~~FP~~PF: Poste forestier (forestry post); OHNP: One Health National Platform; SS: sanitary services; TFP: technical and financial partners; TS: technical secretary; ~~VP~~PV: Poste vétérinaire (Veterinary post); ZATE: Zone d'Appui Technique à l'Elevage (Livestock Technical Support Zone).

Formatted Table

Discussion

The co-construction process implemented in Burkina Faso proved fruitful in supporting stakeholders towards the implementation of ~~an~~a One Health ~~integrated anthrax~~ surveillance system for anthrax. It led to collective representation of the current ~~situation~~ surveillance system and of the desired ~~situation~~surveillance system, as well as the definition of changes and actions to move from one to the other. However, because of time constraints, the action plan was not finalised.

Formatted: English (United Kingdom)

Formatted: English (United Kingdom)

In the course of defining One Health~~integrated~~ surveillance, participants from the animal health and environmental sectors demonstrated a good understanding of what surveillance means in a One Health approach, and of the issues involved. Some participants from the human sector, however, were unfamiliar with the concept, and interpreted integrated surveillance as meaning good coordination and collaboration between actors within the same intra-sectoral surveillance programme, operating at different decision-making levels. The definition finally agreed upon by the participants is consistent with those in the literature for integrated surveillance in a One Health approach [17, 13]. The co-construction process, by providing a framework for participants to compare their perspectives, highlighted several points of divergence but also enabled participants to engage in a learning process towards greater mutual understanding.

The quality of a participatory process significantly impacts the quality of its results [18]. The ability of any participatory process to produce relevant results is based on several elements: (i) the representativeness, knowledge and influence ~~capacities~~ of the participants; (ii) the ability of the method to elicit, compile and synthesize information from different sources to create representations to which participants can reasonably adhere; and (iii) the quality of the facilitation [15]. The results produced by the participatory process must therefore be analysed in the light of the quality of the process itself.

Despite our efforts to bring together a panel of participants who are representative of the key actors in the anthrax surveillance system in Burkina Faso, it was difficult to benefit from the presence of certain actors and/or maintain their commitment throughout the workshop. Moreover, it was not just a question of having all categories of stakeholders represented, but also of the representative sent by the invited institution being relevant to the workshop's objective. This was not the case for the representative of the animal health sector at local level, who had neither a role in nor experience of anthrax surveillance. There is also the question of how representative a participant's point of view is of the institution represented. Indeed, points of view can diverge from one individual to another within the same category of stakeholders, and inviting a single representative from a given institution to this type of process is likely to be insufficient to capture and consider exhaustively the knowledge and expectations of that category [9, 19]. Finally, the ability of the process to generate change outside its arena is strongly linked to the participants’ power in influencing decision-making. In our case, where the main objective was to produce an action plan, the question of its implementation arises if the collective decisions taken during the workshop are not taken to a high enough decision-making level [8]. Some categories of stakeholders appeared to be more involved than others in the issue of ~~integrated anthrax~~One Health surveillance. Environmental and animal health participants showed a greater interest than their human health counterparts. There are two possible reasons for this. The first is that ~~integrated~~ One Health surveillance is still viewed in a very anthropocentric way. In this scenario, the workshop thus represented an opportunity for the animal and environmental sectors, which have more limited resources for surveillance than the human sector, to argue in favour of a better distribution of available resources between sectors, and also between levels of intervention [8]. The second is that zoonoses remain a marginal issue for the human health sector, particularly compared with infectious diseases of non-animal origin (such as malaria or HIV) and non-infectious diseases (such as diabetes), which represent a greater health and socio-economic impact [20].

But ensuring that stakeholders are properly represented is not enough to guarantee the success of a participatory process. The challenge then lies in ensuring that a plurality of viewpoints is expressed and combined to generate new knowledge and collective decisions. Managing diversity can be complex, posing implementation difficulties that compromise the achievement of the objectives sought through the process [21]. Furthermore, participatory processes require more commitment and energy from participants than traditional approaches, where participants are more passive [22]. The quality of the methodology adopted and the ability of the facilitation team to implement it are therefore key determinants of the success of a participatory process [15, 18]. The composition of the facilitation team and the quality of facilitation are also critical to the success of such a process, as the facilitator or facilitators hold the keys to the change process [11]. On the one hand, it is important to have people in that role who will moderate in an almost "naïve" way and not bring their own opinion to bear on what participants say. On the other hand, it can be beneficial for facilitators to have some expertise in the field, to enable them to challenge participants on potential inconsistencies or areas that may not have been addressed [15]. Indeed, it is not certain that the mere confrontation of different points of view will lead to an accurate analysis of the situation and needs, let alone to a compromise. The facilitator therefore also has a role to play in sharing and debating objective information [11]. In addition, the facilitator's role is to ensure that the plurality of viewpoints is considered. To do this, the facilitator must be able to manage conflicts and power dynamics that may represent an obstacle to the collective process [23]. For our workshop, the facilitation team was diverse, including contextual, technical, and methodological experts.

Despite all the efforts to develop participatory processes that are inclusive, adapted to the context and adaptive, participatory processes are complex and influenced by social and political factors that are beyond the control of the process initiators [10].

The power relationships between institutions and stakeholder categories outside the participatory forum are likely to be reproduced within the process, and some participants are likely to have a greater

influence than others on the decisions taken. There is therefore a risk that the participatory process may actually reinforce power imbalances by presenting its results as the fruit of a collective reflection, while the contributions from actors of different categories may have been unequal and the results may in fact mainly reflect the concerns of the actors from dominant categories [24]. During our workshop, for example, the hierarchical position and high social recognition of some participants sometimes hampered the free debate of ideas and sharing of perspectives. Moreover, the act alone of expressing their point of view publicly does not guarantee that stakeholders from categories with less power will truly be listened to and heard. Indeed, where there are tensions, free expression by participants can even have the opposite effect, by reinforcing those points of tension and power imbalances [11].

In the same vein, the decisions that will be taken, even in a collective and concerted manner, as part of the participatory process, have no certainty of success because they are dependent on elements external to the process [12]. In the case of our participatory process, most proposed actions are dependent on the availability of a sufficient budget, itself largely dependent on funding granted by technical and financial partners, who may have different priorities from those that guided the action plan. Implementation of the action plan co-constructed with the stakeholders therefore lies not so much in the changes engendered in the surveillance stakeholders as in changes in the rules of the game that determine decision-making and financial flows [11].

Finally, despite the quality of the method and facilitation, in some cases it may be difficult to engage certain individuals who have cultural values and cognitive framework that hinder their participation in this type of co-construction process that is based on listening, sharing, and co-learning [10].

The co-construction process implemented in Burkina Faso took place over a three-day workshop. It would have benefited from being more iterative, through the organization of further workshops during which participants could have reflected on the results produced, to discuss and develop them further [14, 25]. This would also have been an opportunity to usefully involve certain categories of stakeholders not included during this first one (farmers, veterinarians and medical practitioners). Moreover, in the case of anthrax surveillance, the front-line actors are those working at local level, and it would have

been interesting to organize workshops with a wider range of local actors by decentralizing them to a sub-national level. In addition, it would be interesting to put a figure on the cost of the actions identified to facilitate the search for funding.

The co-construction process proposed in Burkina Faso to collectively define the modalities for implementing the One Health ~~integrated anthrax~~ surveillance system for anthrax appears satisfactory in terms of the results produced and the active participation of those invited. Participants expressed their satisfaction regarding the workshop organization and the results produced, and stated that they would take actions to contribute to the application of the decisions taken. They also considered that they improved their knowledge of the epidemiology of anthrax, as well as of the organization and functioning of anthrax surveillance. However, this evaluation remains highly subjective. Despite the anticipated benefits, participatory processes are still largely controversial. They are often criticized by decision-makers as costly in terms of time and resources [25]. They are often questioned because the results produced are very largely dependent on the quality of facilitation and the representativeness of participants [14]. Additionally, from our operational perspective, the results produced cannot claim to be the most suitable and appropriate for the simple reason that they were co-constructed, since they are representative of only part of the viewpoints and were influenced by the social and political context in which they were expressed [10]. It is therefore necessary to explicitly document the implementation of the process, to assess its intrinsic quality (method used, facilitation, representativeness of participants) and to measure its effects (changes engendered among participants, or on the implementation context) in a robust way, while considering other factors that could also have contributed to the observed effects [13]. Monitoring and evaluating the participatory process is also an opportunity to highlight elements that may impact the quality and effects of the process and therefore redirect the course of the process to take these elements into account [12].

Conclusions

The process of co-constructing ~~an integrated~~ a One Health ~~anthrax~~ surveillance system for anthrax in Burkina Faso enabled representatives of surveillance stakeholders to collectively define their vision of the ideal One Health surveillance system for anthrax. They were then able to establish the path to be taken from the current situation to this desired vision of anthrax surveillance. This co-construction process was also an opportunity for the participants to engage in a process of technical and social learning, by exchanging their points of view on the problem and the solutions for dealing with it. This pooling of knowledge, which generates new collective knowledge, creates a conducive environment for making collective decisions and planning future action. The interactions that take place between participants during the process provide the basis for new partnerships and collaboration, conducive to the implementation of more integrated health policies.

The methodological framework we developed and used to conduct the participatory process consists in four key steps: defining the ideal situation or vision of the future; characterizing the current situation; identifying changes to achieve the ideal situation; and drafting an action plan to operationalize the changes. However, the implementation of the method needs to be adaptive and iterative, adapting along the way not only to the knowledge shared by the participants, but also to the posture of the participants in relation to the approach and to each other. The adopted method and the quality of facilitation is therefore a key element in the success of the participatory process.

Each participatory process is unique because it is specific to the context in which it is implemented, the participants mobilized, and the objectives pursued [12]. However, our study proposes a methodological framework that can be used, after adaptation, in other contexts to address different issues.

Acknowledgements

The authors would like to thank all the participants who agreed to take part in the participatory process.

References

1. World Health Organization: Tripartite and UNEP support OHHLEP's definition of "One Health": Joint Tripartite (FAO, OIE, WHO) and UNEP Statement. 2021. https://www.who.int/news/item/01-12-2021-tripartite-and-unep-support-ohhlep-sdefinition-of-one-health. Accessed 21 October 2023.
2. Hayman DTS, Adisasmito WB, Almuhairi S, Behravesh CB, Bilivogui P, Bukachi SA, et al. Developing One Health surveillance systems. One Health. 2023;100617.
3. Babo Martins S, Rushton J, Stärk KDC. Economic assessment of zoonoses surveillance in a 'One Health' context: a conceptual framework. Zoonoses Public Health. 2016; 63: 386–95.
4. Aenishaenslin C, Häsler B, Ravel A, Parmley J, Stärk K, Buckeridge D. Evidence needed for antimicrobial resistance surveillance systems. Bull World Health. 2019; 97(4): 283–9.
5. Zinsstag J, Utzinger J, Probst-Hensch N, Shan L, Zhou XN. Towards integrated surveillance-response systems for the prevention of future pandemics. Infect Dis Poverty. 2020; 9(1): 140.
6. Bennani H, Cornelsen L, Stärk KDC, Häsler B. Evaluating Integrated Surveillance for Antimicrobial Use and Resistance in England: A Qualitative Study. Front Vet Sci. 2021; 8: 743857.
7. Streichert LC, Sepe LP, Jokelainen P, Stroud CM, Berezowski J, Del Rio Vilas VJ. Participation in One Health Networks and Involvement in the COVID-19 Pandemic Response: A Global Study. Front Public Health.2022; 10: 830893.
8. Bordier M, Uea-Anuwong T, Binot A, Hendrikx P, Goutard FL. Characteristics of One Health surveillance systems: A systematic literature review. Preventive Veterinary Medicine. 2020; 181: 104560.

Field Code Changed

9. Nana SD, Caffin JH, Duboz R, Antoine-Moussiaux N, Binot A, Diagbouga PS et al. Towards an integrated surveillance of zoonotic diseases in Burkina Faso: the case of anthrax. BMC Public Health. 2022; 22(1): 1535.
10. Lavigne Delville P, Mathieu M. Donner corps aux ambitions : le diagnostic participatif comme enjeu de pouvoir et comme processus social, in Lavigne Delville P, Sellamna NE, Mathieu M Les enquêtes participatives en débat : ambitions, pratiques et enjeux. Paris : Karthala, 2000. Article in French
11. Rowe G, Frewer LJ. Evaluating public-participation exercises: a research agenda. Science, technology, and human values. 2004; 29(4): 512-556.
12. Hassenforder E. Participatory planning, evaluation, and institutional dynamics in African natural resource management. Australian National Universit. 2015.
13. Bordier M, Delavenne C, Nguyen DTT, Goutard FL, Hendrikx P. One Health Surveillance: A Matrix to Evaluate Multisectoral Collaboration. Front Vet Sci. 2019; 6: 109.
14. Groot A and Maarleveld M. Demystifying facilitation in participatory development. IIED, London. 2000.
15. Etienne M. Companion Modelling. A Participatory Approach to Support Sustainable Development. Versailles: QUAE. 2011
16. Phdungsilp A. Futures studies' backcasting method used for strategic sustainable city planning. Futures. 2011; 43(7): 707-714
17. Stärk KDC, Arroyo Kuribreña M, Dauphin G, Vokaty S, Ward MP, Wieland B, et al. One Health surveillance – More than a buzz word? Preventive Veterinary Medicine. 2015; 120(1): 124
18. Glicken J. Getting stakeholder participation 'right': a discussion of participatory processes and possible pitfalls. Environmental Science and Policy. 2000; 3(6): 305-310.
19. Grünenfelder J and Schurr C. Intersectionality – a challenge for development research and practice? Dev. Pract. 2015; 25 (6): 771–784.

20. World Health Organization: Integrating neglected tropical diseases into global health and development: fourth WHO report on neglected tropical diseases. 2017. https://iris.who.int/bitstream/handle/10665/255011/9789241565448-eng.pdf?sequence=1. Accessed 4 August 2023.

21. . 2017s2017. Accessed 4 August 2023.

22. Weaver L and Cousins JB. Unpacking the participatory process. Journal of Multidisciplinary evaluation. 2004; 1(1): 19-40.

23. Mansuri G and Rao V. Community-based and-driven development: A critical review. The World Bank Research Observer. 2004 ;19(1): 1-39.

24. Pretty JN, Guijt I, Thompson J, Scoones I. Participatory Learning and Action: A Trainer's Guide. London: The International Institute for Environmental and Development. 1995.

25. Lee K, Brumme ZL. Operationalizing the One Health approach: the global governance challenges. Health Policy and Planning. 2013; 28(7): 778

26. Binot A, Duboz R, Promburom P, Phimpraphai W, Cappelle J, Lajaunie C, et al. A framework to promote collective action within the One Health community of practice: Using participatory modelling to enable interdisciplinary, cross-sectoral, and multi-level integration. One Health. 2015; 1: 44

Field Code Changed

Responses to reviewer comments

REVIEWER 1

Introduction

The authors don't mention the presence of a One Health National Platform in Burkina Faso. So, among the participant invited, this stakeholder is don't take account. Even if another paper is already published on the current involved stakeholders in the anthrax surveillance, it is necessary to remind briefly here.
We have mentioned the platform in the introduction of the revised manuscript (see lines 51 and 52)

Material and methods

In the participatory process, it is necessary to implement the system and use results to correct the system. That can constitute the fifth step.
In participatory planning, the participatory process aims at the co-construction of an action plan for the establishment of the desired One Health surveillance system. The implementation of the action plan is not part of the process.
However, it is valuable to evaluate the implementation of the action plan and its effects, as part of the evaluation process of the participatory process. We developed this point in lines 380-386.

When you say Central and local authorities, it is necessary to precise the level of the positioning (National, provincial, departmental, village, etc.). In the table 2, at the points 5.2 & 5.3, you mentioned Regional, provincial and local level. What "local level" means?
By local authorities, we refer to State agents working in the field, i.e the ones who are at the frontline for the observation of the health events. Depending on their supervising ministry, their area of action differs but their level is below the provincial one. So, we cannot specify a generic administrative level suitable for all the local authorities. We have revised the manuscript to clarify this point, as followed: "Local authorities refer to field actors working for ministries in charge of public health, animal health and wildlife conservation" (see lines 96-97).

For each institution, what kind of personal (head or agent) was took account? That is important in the future implementation of the changes' actions.
The target audience was technical agents directly involved in surveillance activities because we assumed that they would be the most capable to share knowledge about surveillance and to identify necessary changes to improve integrated surveillance. The drawback of this approach is that the changes and actions defined are not communicated and bought in by the people who have the power to implement them. This is a limitation of our study that we discussed lines 296-299.

From the Table 1, can you explain the reason of the total or partial absence of some stakeholder? What were the financial supported for the participants? That can explain also the quality of the participants.
The two missing participants were engaged in other official duties on these days. One missed only one on the three days (the 2nd day), and this suggests his interest for the workshop.
Participants were compensated for their travel expenses and were offered lunch for the three days. Those travelling from provincial areas were compensated for accommodation and food expenses.
Providing financial support may also have a negative impact on the quality of the participants, as some people may be attracted by the money, more than by the interest for the topic. In our study, we did not use financial support to motivate people but we created a climate of trust with participants with whom we had interacted prior the workshop. We also paid attention to the date and location of the workshop to improve the likelihood of their availability and attendance (see lines 97-99).

Lines 98 – 99: "appropriate participative tools" used can be cited here. Tools used are so important as the quality of facilitators.
The participative tools used and the way they were used are described at each step of the participatory process in the Material and Method part.

Lines 104 – 106: The evaluation is an essential part of the process building. The evaluation method must be presented in this paper.
Because of word limitation, we cannot present the evaluation plan in this paper but we intend to publish it in a separate paper. However, we have added a short description of it: "Qualitative and semi-quantitative indicators were measured based on data collected before, during and after the workshop, using different methods (questionnaire, semi-structured interviews, workshop observation)" (see lines 111-113).

Figure 2: Why different boxes has the same number? The numbers must be different and classified. Review also the abscissa axis texts: What mean T0? T0 + 10 ***ans****?*
The figure has been updated following the recommendations and a legend added. Additionally, "ans" has been changed for "years".

RESULTS

Line 181: notification of suspected or confirmed cases? Need the precision. In the lines 201 to 207, it is mentioned the suspected cases.
The notification of suspected cases must be followed by an investigation to confirm or infirm them. We have updated the wording of the title to be consistent with the changes identified (see line 213).

How, the time 10 years had been defined? Is it the time of evaluation of the impact?
The time step was defined on the basis of our knowledge of the surveillance context in Burkina Faso. It seemed to us to be the most realistic for setting up an integrated surveillance system. Far enough away for participants to be able to propose far-reaching changes. Not too far in the future for them to be able to project themselves. This timeframe was not questioned by the participants, suggesting that they were also comfortable with this choice. We have made this precision in the revised manuscript (see lines 123-125).

Figure 3, 4, 5, 6 & 7. Review pictures' resolutions for a best reading
Figures resolution has been improved and checked using the PLOS tool. Please note that when the PLOS system builds the PDF which is sent to reviewers, the resolution is decreased. To see the original file, you need to click on "Click here to access/download xxx" at the top of the page where the figure is displayed.

Table 2: Repeat the columns' titles in each page can help for the best reading du to the width of this table.
The table has been updated to meet this request.

In the point 7 of the table 2, keep the original denomination of all services: ZATE, CSPS, PV, PF, etc.)
The table has been updated in the revised version of the manuscript to take into consideration this comment.

The action plan presented in the table 2 is not planed in time. How that can be reached before the 10 years?

During the workshop, we only worked in defining the staring time and time period for the first action of each change. We did not include it in the manuscript for space reason and because we consider that this information was not crucial in a scientific article.

At this stage of your study, is it possible to know if process succeed or not? That is not clear in the manuscript.
That depends what we mean by success. This question will be partially answered with the results of the evaluation process that will be published in a separate manuscript. Indeed, so far, only the immediate and intermediate effects of the process have been evaluated. Long-term effects, including the implementation of action plan and its impacts, have not been evaluated yet. It is important to mention that the success of a participatory process does not only rely in the results produced but also in the knowledge and mutual understanding gained by participants during the process.
We know that the action plan has not been approved yet but that it will be used to develop the upcoming surveillance action plan piloted by the national One Health Platform.
We have updated the manuscript to include some observations that suggested that the workshop was a success regarding some criteria: "The co-construction process proposed in Burkina Faso to collectively define the modalities for implementing the One Health surveillance system of anthrax appears satisfactory in terms of the results produced and the active participation of those invited. Participants expressed their satisfaction regarding the workshop organization and the results produced, and stated that they would take actions to contribute to the application of the decisions taken. They also considered that they improved their knowledge about the epidemiology of anthrax, the organization and functioning of anthrax surveillance" (see lines 369-373).

Discussion

Is it possible to evaluate the cost of the changes proposed to improve the current surveillance system? Also discuss the feasibility of this changes regarding the cost, and the financial sources.
An economic evaluation would be a strong added value to support the implementation of the action plan but it was not conducted in this study. This perspective has been added in the discussion part of the manuscript (see lines 364-365).
Allocation of appropriate financial resources has been identified a main barrier to the implementation of the action plan and this has been added in the revised manuscript (see lines 254-255).

REVIEWER 2

Title:

This can be read as a parallel system along with the existing sectoral ones. Which is not the case, refer to. figure 4. The word "current" is used in the article to talk about the sector surveillance system, however. Make it i explicit and clear that the One Health anthrax surveillance "system" is developed by leveraging the existing sector surveillance system and creating bridges between them.
We proposed to change the initial title to:
"A participatory approach to move towards a One Health surveillance system for anthrax in Burkina Faso". (See lines 1-3).
Moving towards a One Health surveillance system is not only creating bridged between sectoral components but also reinforcing sectoral surveillance performance (as underlined by participants of this study).
In the introduction, we mentioned explicitly that we were not proposing to develop a new system but to improve the current one, in line with the OH concept: "identify the changes, and associated

actions, necessary to evolve from the current situation to the desired situation" (see lines 74-76). We make it more explicit in the Materials and Methods section (see lines 83-84).

Abstract:

IS this a definition of an integrated surveillance system through One Health lens?
We use the term integration in its primary signification, which is "to bring together or incorporate (parts) into a whole". In the context of surveillance, integration refers to the fact different that surveillance components are linked together. In a One Health context, integrated surveillance brings together surveillance components that are operating in the three main sectors (when relevant): human, animal, environment.
We made the definition more precise: "Regarding epidemiological surveillance, this implies the development of integrated systems that link surveillance components operating in different domains (human, domestic animals, environment) and involving several actor networks." (see lines 20-22).

Be specific, add the type of interaction, Is it for instance information sharing?
Interaction may happen both at governance (steering, coordination, technical and scientific support) and operation levels (data collection, data management and storage, data analysis and interpretation, result communication and dissemination).
We have updated the manuscript as follow: "However, surveillance continue to operate in a very compartmentalized way, with little interaction between sectoral institutions and with the community for the governance and operation of surveillance activities." (see lines 22-25).

I suggest to rewrite this sentence. This can be read as an affirmation, which stakeholders are you referring to?
This is well documented that development and research programmes are usually designed and implemented with an insufficient consideration of the points of view of all categories of stakeholders, especially actors working at the local level.
However, in order to balance our words, we proposed to rewrite the sentence, as followed:
"This is partly explained by the insufficient consideration of the local context and the late involvement of national stakeholders when developing programmes that aimed at strengthening the integration of surveillance." (See lines 25-27).

This can be read as national counterpart does not have a say. If correct, I suggest to rewrite this sentence to have a better balance
The manuscript is mentioning the influence of the external partners (through the organization and financing of workshops, seminars and trainings in particular). This does not suggest that national actors cannot raise their voice. We think that the current wordings is correct to describe this situation.

[Mutual understanding] of what? the existing systems?, if yes, be specific
We have defined this term in the introduction (lines 47-48): *understanding one another's thoughts, feelings, and perspectives)*

What about coordination and cooperation? [in addition to collaboration]
Collaboration is at the heart of the One Health concept as mentioned in most of the definitions of the concept in the literature. We use collaboration in a comprehensive way that covers also collaboration for coordinating activities and for communication. Cooperation is a very close concept to collaboration. Adding coordination and cooperation will make the text more difficult to read, without bringing crucial additional information.

Introduction

[Regarding non tangible benefits] Can be read as non-monetary. if correct, use non-monetary
Non tangible benefits, as well as tangible benefits, could be monetary and non-monetary. So non tangible is not synonymous with non-monetary.

Materials and methods

[Regarding the participants] Which quality? is related to the Knoweledge on the matter/subject?
The quality of participants is not referring to the knowledge only. It is related to the adequation between the participant characteristics (in terms of capacities, capabilities, knowledge, practices, etc.) and the category of the stakeholder he/her is representing

[Regarding participant selection], I understand, selection criteria was applied. Correct?
Yes, as described right after.

I understand, a consensus on the defined vision, correct?
In this type of approach, we are not necessarily looking for a consensus; it may sometimes be a question of compromise, the objective being that all the participants find themselves in the representation produced

evaluation matrix or evaluation plan?
This an evaluation plan

Is it for each sector? If yes, be specific.
No, it is not. It is the whole surveillance system, ie all actors involved in surveillance, whatever their sector, and interactions between them (within a sectoral surveillance component and between sectoral components). We believe that the sentences just after clarify already this point.

The next two comments were uncompleted sentences and could not be addressed:
"This can be read as an assessment (formal or not) of existing surveillance systems was con"
"Is the information flow w"

Is any identified official document used to "which actor needs to do what differently?
We did not explicitly refer to any official document to support this step. We let participants expressed changes based on their knowledge and expectations, which may have been influenced by official documents, such as international standards or national legal instruments. But, we did not ask and record for this information.
Expressing changes in terms of "which actor needs to do what differently?" is the most commonly adopted way in change-oriented approaches.

[Regarding the use of integrated surveillance], use One Health surveillance instead
We changed integrated surveillance system to One Health surveillance system, where appropriate.

Results

[Regarding the formalization of exchanges] *This can be read as there is no administrative decision of their involvement as actor. Is it correct?*

This means that there is no official document describing the terms of these exchanges. We have revised the manuscript to make this clearer: “All these changes were made with a high degree of consensus, except for the interactions linked to information sharing between field actors, which gave rise to differences of opinion. In fact, these exchanges are not formalized in an official document, …”. (See line 202-206)

[Regarding changes proposed by the participants] *If there is no administrative decision/adopted formal framework or any other formal documents to streamline multisectoral collaboration, coordination and cooperation acheiving these changes might be difficult*
Is this disccused during the workshop?
The participants discussed this point. That’s why they identified some changes in terms of intersectoral governance (see lines 235-238) and the need to validate and implement the action plan through the inter-ministerial platform (see lines 253-254).

Suggestion to change “A specific intersectoral plan” for “A anthrax intersectoral actionable plan (WHAT, WHEN, HOW, WHO,....) should be developed and implemented.”
This paper reflects the views of the workshop participants who expressed themselves with this terminology. Consequently, we propose to keep the text as it is.

What is the added value of integrating the anthrax specific intersectoral plan into the global intersectoral plan for zoonoses
The participants thought that the plan will gain in attention if it was not staying standalone but integrated in an overarching document, making it easier to seek funding for initiatives. Moreover, some actions might be applicable for other diseases and thematic. We have updated the manuscript to mention this point (see lines 238-240).

You should propose to conduct a multisectoral After Action Review when a Anthrax event is closed
This paper reports changes and actions that were proposed by workshop participants. It is not intended to reflect the view of the researchers who facilitated the workshop.

Above, the word "report" was used. Use the same word here and accross the manuscript
We changed report (fewer occurrences) for notification in the whole manuscript.

Is the OHP invited aven as observer?
The OHP was one of the participants of the workshop, as mentioned in table 1 (national authorities at the multisectoral level).

[Regarding the lack of political will], Be more explicite? What do you mean by this?
We refer to the lack of political will to allocate resources for surveillance. We brought this precision in the manuscript: “The existing national One Health platform represents an appropriate governance framework for implementing the actions identified. However, the lack of political will to allocate more resources for surveillance” (see line 255).

Regarding request to change order in table 2.
We kept changes in the chronological order as defined by participants during the workshop. They considered that equipping agents with tablets was coming first, then updating the current forms, and finally training the agents. This can be discussed and revised at a next step of the process but for now, for ethical reason, we prefer to strictly comply with what was agreed on during the workshop.

Discussion

[Regarding the collective representation of the current situation] *be specific. of what? Sector surveillance system? This wording can be understood as an assessment was conducted. The fig 4 provides information flow within each system and the arrows the necessary bridges among the systems.*
This is a representation of the current situation of the surveillance in Burkina, in all sectors combined. We made this clearer in the revised manuscript: "It led to a collective representation of the current surveillance system and the desired surveillance system" (see lines 264-265).
We fail to see how this wording can suggest that an assessment was done. A representation is a way to model a situation, in our case, through the design of an actor diagram. Figure 4, as mentioned in the title, is a representation of the current surveillance situation (in all sectors) using an actor diagram, where actors are represented together with the interactions that exist between them (are they belonging to the same sectoral component or not).

Instead of capacity use the knowledge and experience on the matter/subject/
Capacities is not only related to knowledge and experience, but also related to representativeness and influence, as developed in the next paragraph.
We updated the manuscript as followed: "(i) the representativeness, knowledge and influence of the participants" (see lines 280-281).

Figure 1

Add s at system
There is only one system, consisting of several surveillance components; so no s is needed at system

add before changes, the words " institutional and technical " changes. Example of Institutional change: adoption of formal framework for information sharing among sector. Example of technical change: Use of koint SOPs,...........
We do not know in advance what kind of changes will be identified by participants and identified changes may not fall into the institutional and technical categories (behavioural changes for instance). As this method is intended to be generic, we prefer to keep changes without any specific qualification.

Photo 2

Add legend. Blue:.....Green:.....Orange: Arrow: …
Is the different colors represent each and the arrows the information flow within and between sectors?
This photo here is to illustrate the participatory tool that was used to support participants in the co-construction process of the current surveillance situation (material and method section). We do not expect the reader to be able to read the actor diagram here. Moreover, it is presented in the result section (with the appropriate legend).

Figure 3.

Community health workers can be read as human health worker. If it is not the case be specific.
It refers to community agents working in the human (CHW), animal (CAHWs) and environmental (ecoguard) sectors. We have changed for community agents to be more generic.

We have updated the figure with the 2 requests of rewording (effective instead of good and addition of administrative before mechanisms)
