# Supplementary material for: A participatory approach to move towards a One Health surveillance system for anthrax in Burkina Faso
Source: PLoS One. 2024 Jun 5;19(6):e0304872. doi: 10.1371/journal.pone.0304872 (PMC11152267; doi:10.1371/journal.pone.0304872)
Supplement: Attachment — Submitted filename: Review.docx [file pone.0304872.s002.docx]

**PONE-D-23-41971**

A participatory approach to support the development of a One Health surveillance system for anthrax in Burkina Faso

This manuscript reports the results of a co-construction approach to develop a One Health surveillance system for anthrax in Burkina Faso. Some actions are proposed by the participants to improve the current surveillance system.

Albeit some minor mistakes, the manuscript is well written. The methods are appropriate for the objectives and the conclusion is supported by the result. I provide here comments for the author`s consideration.

**Introduction**

The authors don’t mention the presence of a One Health National Platform in Burkina Faso. So, among the participant invited, this stakeholder is don't take account. Even if another paper is already published on the current involved stakeholders in the anthrax surveillance, it is necessary to remind briefly here.

**Material and methods:**

In the participatory process, it is necessary to implement the system and use results to correct the system. That can constitute the fifth step.

When you say Central and local authorities, it is necessary to precise the level of the positioning (National, provincial, departmental, village, etc.). In the table 2, at the points 5.2 & 5.3, you mentioned Regional, provincial and local level. What “local level” means?

For each institution, what kind of personal (head or agent) was took account? That is important in the future implementation of the changes’ actions.

From the Table 1, can you explain the reason of the total or partial absence of some stakeholder? What were the financial supported for the participants? That can explain also the quality of the participants.

Lines 98 – 99: “appropriate participative tools” used can be cited here. Tools used are so important as the quality of facilitators.

Lines 104 – 106: The evaluation is an essential part of the process building. The evaluation method must be presented in this paper.

Figure 2: Why different boxes has the same number? The numbers must be different and classified.

Review also the abscissa axis texts: What mean T0? T0 + 10 **ans**?

**RESULTS**

Line 181: notification of suspected or confirmed cases? Need the precision. In the lines 201 to 207, it is mentioned the suspected cases.

How, the time 10 years had been defined? Is it the time of evaluation of the impact?

Figure 3, 4, 5, 6 & 7. Review pictures’ resolutions for a best reading.

Table 2: Repeat the columns’ titles in each page can help for the best reading du to the width of this table.

In the point 7 of the table 2, keep the original denomination of all services: ZATE, CSPS, PV, PF, etc.)

The action plan presented in the table 2 is not planed in time. How that can be reached before the 10 years?

At this stage of your study, is it possible to know if process succeed or not? That is not clear in the manuscript.

**Discussion**

Is it possible to evaluate the cost of the changes proposed to improve the current surveillance system? Also discuss the feasibility of this changes regarding the cost, and the financial sources.
